# Effects of transcutaneous neuromuscular electrical stimulation on post-stroke dysphagia: a systematic review and meta-analysis

**DOI:** 10.3389/fneur.2023.1163045

**Published:** 2023-05-09

**Authors:** Yuhan Wang, Lu Xu, Linjia Wang, Minjiao Jiang, Ling Zhao

**Affiliations:** ^1^Acupuncture and Moxibustion College, Chengdu University of Traditional Chinese Medicine, Chengdu, Sichuan, China; ^2^Gastroenterology Department, Yongchuan Traditional Chinese Medicine Hospital Affiliated to Chongqing Medical University, Chongqing, China; ^3^Acupuncture and Moxibustion College, Nanjing University of Traditional Chinese Medicine, Nanjing, Jiangsu, China

**Keywords:** stroke, dysphagia, neuromuscular electrical stimulation, meta-analysis, evidence-based medicine

## Abstract

**Background:**

Dysphagia is one of the common complications after stroke. It is closely related to lung infection and malnutrition. Neuromuscular electrical stimulation (NMES) is widely used in the treatment of post-stroke dysphagia, but the evidence-based medical evidence of NMES is limited. Therefore, this study aimed to evaluate the clinical efficacy of NMES in patients with post-stroke dysphagia by systematic review and meta-analysis.

**Methods:**

We searched the CNKI, Wanfang, VIP, SinoMed, PubMed, Embase, Cochrane Library, and Web of Science databases for all randomized controlled trials (RCTs) of NMES in the treatment of post-stroke dysphagia from the establishment of the database to 9 June 2022. The risk of bias assessment tool recommended by Cochrane and the GRADE method was used to assess the risk of bias and the quality of evidence. RevMan 5.3 was used for statistical analysis. Sensitivity and subgroup analyses were performed to evaluate the intervention effect more specifically.

**Results:**

A total of 46 RCTs and 3,346 patients with post-stroke dysphagia were included in this study. Our meta-analysis showed that NMES combined with routine swallowing therapy (ST) could effectively improve swallowing function in Penetration-Aspiration Scale (MD = −0.63, 95% CI [−1.15, −0.12], *P* = 0.01), Functional Oral Intake Scale (MD = 1.32, 95% CI [0.81, 1.83], *P* < 0.00001), Functional Dysphagia Scale (MD = − 8.81, 95% CI [−16.48, −1.15], *P* = 0.02), the Standardized Swallowing Assessment (MD = −6.39, 95% CI [−6.56, −6.22], *P* < 0.00001), the Videofluoroscopic Swallow Study (MD = 1.42, 95% CI [1.28, 1.57], *P* < 0.00001) and the Water swallow test (MD = −0.78, 95% CI [−0.84, −0.73], *P* < 0.00001). Furthermore, it could improve the quality of life (MD = 11.90, 95% CI [11.10, 12.70], *P* < 0.00001), increase the upward movement distance of hyoid bone (MD = 2.84, 95% CI [2.28, 3.40], *P* < 0.00001) and the forward movement distance of hyoid bone (MD = 4.28, 95% CI [3.93, 4.64], *P* < 0.00001), reduce the rate of complications (OR = 0.37, 95%CI [0.24, 0.57], *P* < 0.00001). Subgroup analyses showed that NMES+ST was more effective at 25 Hz, 7 mA or 0–15 mA, and at courses ( ≤ 4 weeks). Moreover, patients with an onset of fewer than 20 days and those older than 60 years appear to have more positive effects after treatment.

**Conclusion:**

NMES combined with ST could effectively increase the forward and upward movement distance of the hyoid bone, improve the quality of life, reduce the rate of complications, and improve the swallowing function of patients with post-stroke dysphagia. However, its safety needs to be further confirmed.

**Systematic review registration:**

https://www.crd.york.ac.uk/PROSPERO, identifier: CRD42022368416.

## 1. Introduction

Stroke has become the second leading cause of death and the first leading cause of disability worldwide due to its high morbidity, disability, and mortality (1, 2). Moreover, some studies have shown that dysphagia is one of the most common complications in stroke patients. Approximately 37%−78% of stroke patients have dysphagia (3). The clinical manifestations of dysphagia include swallowing disorder, drinking cough, salivation, and other symptoms. Dysphagia after stroke is closely related to malnutrition, dehydration, electrolyte disorder, pulmonary infection, anxiety, and depression (4, 5), and it also leads to prolonged hospitalization, decreased quality of life, and further increased risk of death (6). Currently, swallowing therapy (ST) is mainly used for post-stroke dysphagia, including swallowing muscle strength and coordination exercises, posture changes, and diet adjustments (7).

However, the single ST takes a long time and has poor patient compliance, with a limited effect on severe dysphagia. More than 10% of patients have residual swallowing problems after ST (8). Therefore, how to effectively improve the swallowing function of patients, achieve oral feeding, and reduce the rate of complications is of great significance (4).

Neuromuscular electrical stimulation (NMES) has been a promising treatment for dysphagia in recent years. It can improve swallowing function by stimulating peripheral nerves to trigger swallowing muscle contraction, promote motor cortex repair, and enhance motor relearning ability (9).

Although some reviews claimed that NMES contributed to the rehabilitation of patients with dysphagia after stroke (10, 11), the number of evaluation measures used in these reviews is small, the number of included trials is limited, and the frequency, current intensity, and duration of electrical stimulation are not explored. Therefore, our study conducted a meta-analysis of the clinical efficacy of NMES in the treatment of post-stroke dysphagia in recent years to further provide valuable guidance and evidence-based medical evidence for the clinical use of NMES in the treatment of post-stroke dysphagia.

## 2. Methods

### 2.1. Protocol and registration

This study followed the guidelines of the Preferred Reporting Items for Systematic Reviews and Meta-analyses (PRISMA) statement (12), and the protocol has been registered with PROSPERO (Registration number: CRD42022368416).

### 2.2. Data sources and search strategy

We searched eight scientific databases, namely, CNKI, Wanfang, VIP, SinoMed, PubMed, Embase, Cochrane Library, and Web of Science. The retrieval time was from the establishment of the database to 9 June 2022. There were no restrictions concerning publication source or language. The searched MeSH terms are listed as follows: [“Transcutaneous Electric Stimulation”[MeSH] OR “Percutaneous Electric Nerve Stimulation” OR “Percutaneous Neuromodulation Therapy” OR “TENS” OR “PENS”] AND [“Stroke”[MeSH] OR “cerebral hemorrhage” OR “cerebral ischemia” OR “cerebrovascular disease”] AND [“Dysphagia”[MeSH] OR “Deglutition Disorder” OR “Swallowing Disorder”] AND [“randomized controlled trial”[MeSH] OR “RCT”]. In addition, a supplementary search was conducted for the references included in the literature. Specific information is given in the Supplementary material.

### 2.3. Inclusion criteria

The inclusion criteria for this meta-analysis were as follows: (1) patients with ischemic or hemorrhagic stroke with clear imaging evidence of relevant pathology on magnetic resonance imaging (MRI) or computed tomography (CT); (2) patients with dysphagia after stroke diagnosed by clinical examination; (3) the participant with no other neurological diseases or other dysphagia; and (4) the same ST intervention (acupuncture, transcranial electrical stimulation, and transcranial magnetic stimulation are not included) performed in the experimental and control groups except the experimental group that received NMES.

### 2.4. Exclusion criteria

The exclusion criteria for this meta-analysis were as follows: (1) non-RCT studies, such as cross-sectional studies, case–control studies, case reports, systematic reviews, and animal experiments; (2) studies in which the baseline consistency test was not given; (3) studies with incomplete data, or studies whose full text could not be obtained; and (4) repeatedly published articles.

### 2.5. Outcomes

The outcome indicators were as follows: (1) Functional Oral Intake Scale (FOIS); (2) Penetration-Aspiration Scale (PAS–Fluid); (3) Functional Dysphagia Scale (FDS); (4) the Swallowing Quality of Life questionnaire (SWAL-QOL); (5) the forward movement distance of the hyoid bone (FMHB); (6) the upward movement distance of the hyoid bone (UMHB); (7) the complication rate (CR); (8) the Standardized Swallowing Assessment (SSA); (9) the water swallow test (WST); and (10) the videofluoroscopic swallow study (VFSS).

### 2.6. Data extraction and management

The retrieved literature was imported into EndNote software for unified management. Two researchers (YW and LX) performed literature screening independently according to the proposed inclusion and exclusion criteria. First, we used EndNote to exclude duplicate literature and then conducted the preliminary screening. The two researchers independently read the title of the literature, keywords, and abstracts and initially excluded the documents that did not meet the inclusion criteria; after that, they downloaded and read the full text to determine whether the literature met the inclusion criteria. If necessary, we would contact the original author by mail or phone to obtain undetermined but important information for this study. The researchers independently extracted the data by a pre-designed data extraction form. The data extraction included (1) basic information about the study: research topic, first author, and publication year; (2) the number of cases, intervention, and course of treatment; (3) key elements of bias risk of assessment; and (4) outcome indicators and outcome statistics concerned. If there was any disagreement, it would be referred to a third researcher (LZ) to determine the final result.

### 2.7. Assessment of risk of bias

According to the bias of risk assessment tool recommended by Cochrane (13), the included literature studies were evaluated, including the random sequence production of the literature, the allocation concealment, the implementation of the blind method, whether the blind method was implemented for the result evaluation, the integrity of the result data, whether the results were selectively reported, and whether there were other biases. When the evaluators (YW and LX) had different opinions, they would discuss or ask for a third party (LZ). The risk of bias figure was drawn by RevMan5.3 software.

### 2.8. Data synthesis and statistical analysis

#### 2.8.1. Measurement of therapeutic effects

In this study, odds ratio (OR) with a corresponding 95% confidence interval (CI) was used for binary variable data, and mean difference (MD) was used for continuous variable data.

#### 2.8.2. Assessment of heterogeneity

After extracting and collating relevant data, this study used RevMan5.3 software for data analysis, and then used *I*^2^ statistics and *Q*-test (χ^2^) to assess the heterogeneity of results. The heterogeneity was considered low when *P* > 0.10 and *I*^2^ <50% (14, 15). The heterogeneity was considered high when *P* < 0.10 or *I*^2^ > 50%.

#### 2.8.3. Data synthesis

If the heterogeneity of each group was small (*P* > 0.10, *I*^2^ <50%), the fixed effect model was used. When the heterogeneity was considerable (*P* < 0.10, *I*^2^ > 50%), the random effect model was used after excluding the influence of significant clinical heterogeneity.

#### 2.8.4. Subgroup analysis and sensitivity analysis

When *P* < 0.10 or *I*^2^ > 50% in the χ^2^ test, the source of heterogeneity was identified by extracting eligible articles one by one to make the sensitivity analysis. If not, subgroup analyses would be performed to identify the sources of heterogeneity according to age, duration of disease, duration of treatment, intensity of electrical stimulation, and frequency of electrical stimulation.

#### 2.8.5. Grading of quality of evidence

This study used the Grading of Recommendations Assessment, Development, and Evaluation (GRADE) method (16) to assess the quality of evidence. GRADE divides the quality of evidence into four levels: High quality: further research is unlikely to change our confidence in effect estimates. Medium quality: further research may significantly impact our confidence in effect estimates and may change estimates. Low quality: further research is likely to have a meaningful impact on our confidence in effect estimates and may change estimates. Very low quality: any estimate of the effect is very uncertain (17). Two researchers (YW and LX) independently assessed the quality of the relevant evidence, and a third researcher (LZ) was notified of any disagreement for consultation.

## 3. Result

### 3.1. Literature search results

**Figure 1:**
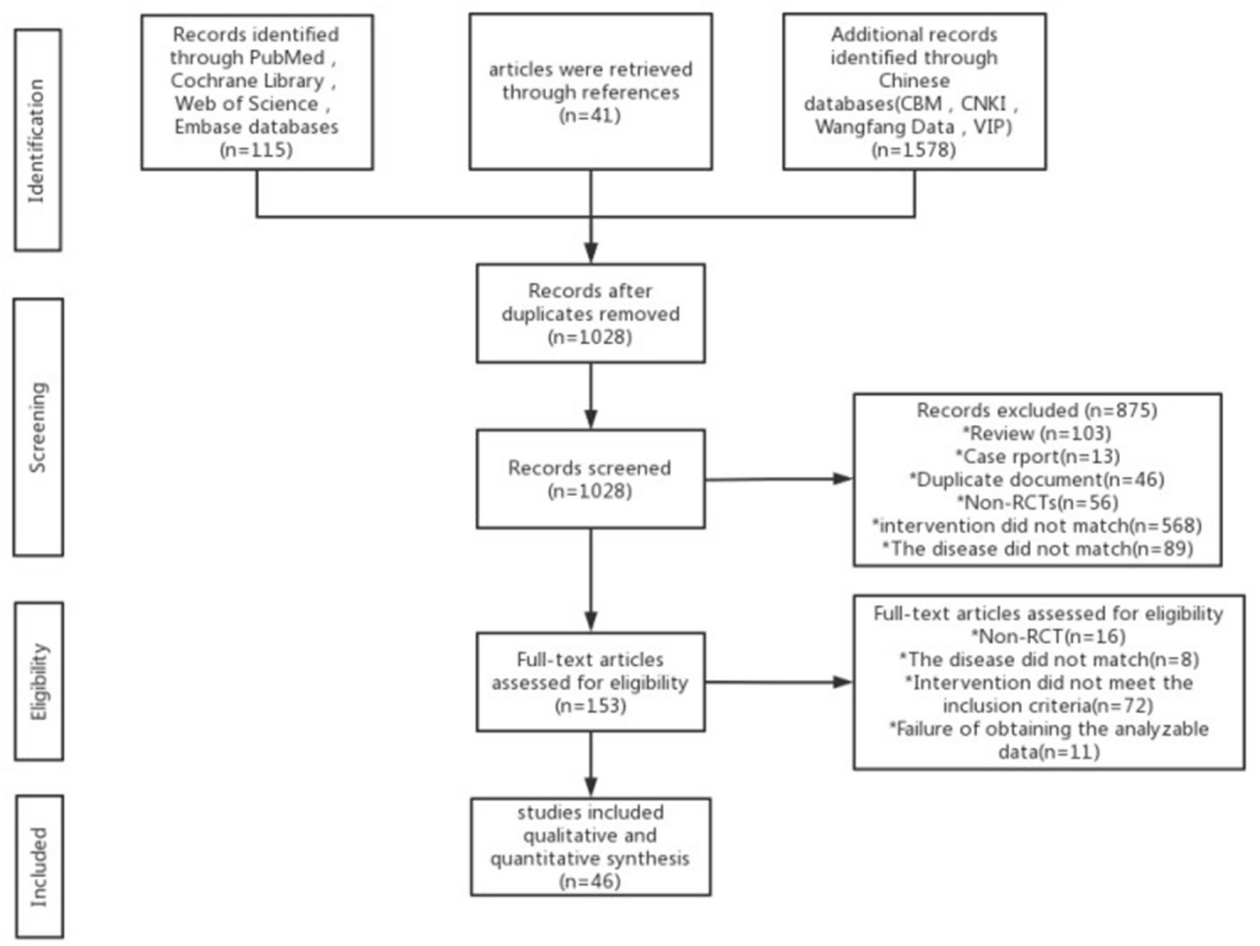
PRISMA flow diagram for study selection.

A total of 1,734 articles were retrieved, and 706 duplicate articles were excluded. After reading the title and abstract, we excluded 875 articles. After reading the complete text, 107 articles were excluded, and 46 articles were included finally. The research selection process is detailed in Figure 1.

### 3.2. Characteristics of the included studies

A total of 46 RCT studies were included, including 3,346 patients with post-stroke dysphagia. Among them, 1,679 patients received NMES + ST, and the other 1,667 received ST. The included studies were from China, the United States, Britain, Italy, Spain, and South Korea, and the treatment course ranged from 2 to 12 weeks, as shown in Table 1.

**Table 1:** Basic characteristics of the included RCTs.

**Study**	**Location**	**Sample size (gender)**		**Age (year)**		**Intervention**		**Period of treatment (week)**	**Outcome measure**
		***T*** **(male/female)**	**C (male/female)**	**T**	**C**	**T**	**C**		
Chang (18)	China	41 (21/20)	42 (23/19)	65.84 ± 6.93	66.43 ± 7.29	NMES + ST	ST	8	1. SSA 2. SWAL-QOL 3. FMHB 4. UMHB
Chen et al. (19)	China	30 (16/14)	30 (15/15)	76.87 ± 9.24	77.04 ± 9.30	NMES + ST	ST	4	1. SSA 2. SWAL-QOL
Chen (20)	China	61 (32/29)	61 (33/28)	53.12 ± 3.43	53.20 ± 3.21	NMES + ST	ST	4	1. SSA 2. SWAL-QOL
Cui et al. (21)	China	63 (38/25)	63 (39/24)	64.12 ± 5.47	64.53 ± 5.36	NMES + ST	ST	2	1. SWAL-QOL 2. VFSS 3. WST
Du and Shao (22)	China	50 (26/24)	50 (27/23)	60.36 ± 2.74	59.25 ± 2.37	NMES + ST	ST	4	1. SSA 2. VFSS 3. WST
Geng (23)	China	71 (38/33)	71 (35/36)	46.78 ± 3.31	46.52 ± 3.26	NMES + ST	ST	4	1. SSA 2. WST
Gong (24)	China	45 (29/16)	45 (28/17)	54.06 ± 17.62	53.06 ± 17.24	NMES + ST	ST	4	1. VFSS
Gu and Shu (25)	China	40 (25/15)	40 (21/19)	71.36 ± 9.23	70.25 ± 8.42	NMES + ST	ST	12	1. SSA 2. SWAL-QOL
Lei et al. (26)	China	56 (30/26)	55 (21/34)	56.15 ± 9.71	54.30 ± 11.34	NMES + ST	ST	2	1. SSA 2. SWAL-QOL
Li (27)	China	40 (22/18)	40 (25/15)	65.72 ± 3.14	66.07 ± 3.27	NMES + ST	ST	8	1. SSA 2. SWAL-QOL 3. UMHB 4. FMHB 5. CR
Liang et al. (28)	China	50 (26/24)	50 (28/22)	62.8 ± 3.2	63.2 ± 2.8	NMES + ST	ST	2	1. SSA 2. WST
Mo et al. (29)	China	41 (31/10)	39 (28/11)	67.13 ± 9.64	65.89 ± 9.23	NMES + ST	ST	4	1. SSA 2. SWAL-QOL 3. CR
Shi et al. (30)	China	60 (33/27)	59 (32/27)	64.98 ± 5.18	65.12 ± 5.14	NMES + ST	ST	4	1. SSA 2. SWAL-QOL 3. VFSS
Tian et al. (31)	China	31 (14/17)	31 (17/14)	51.2 ± 2.3	52.1 ± 3.1	NMES + ST	ST	4	1. SSA
Wang et al. (32)	China	42 (NA)	40 (NA)	NA	NA	NMES + ST	ST	3	3. VFSS
Wang (33)	China	41 (24/17)	41 (23/18)	59.34 ± 8.12	59.52 ± 7.60	NMES + ST	ST	4	1. SSA 2. SWAL-QOL 3. UMHB 4. FMHB
Wang and Ye (34)	China	30 (15/15)	30 (16/14)	67.28 ± 4.75	67.17 ± 4.79	NMES + ST	ST	4	3. VFSS
Wang (35)	China	37 (20/17)	37 (19/18)	61.8 ± 6.5	63.0 ± 7.1	NMES + ST	ST	4	1. SSA 2. SWAL-QOL
Wang et al. (36)	China	30 (17/13)	30 (18/12)	63.6 ± 11.6	62.8 ± 11.3	NMES + ST	ST	4	1. SSA 2. SWAL-QOL
Wen and Wu (37)	China	41 (20/21)	41 (22/19)	68.13 ± 6.74	67.45 ± 7.12	NMES + ST	ST	4	1. SWAL-QOL 2. WST
Zhan et al. (38)	China	24 (13/11)	24 (15/9)	64.3 ± 2.9	65.6 ± 3.1	NMES + ST	ST	2	1. UMHB 2. FMHB
Zhang et al. (39)	China	27 (14/13)	28 (11/17)	63.7 ± 6.3	62.3 ± 8.1	NMES + ST	ST	6	1. CR
Zhang et al. (40)	China	64 (34/30)	64 (35/29)	64.78 ± 5.34	63.91 ± 5.52	NMES + ST	ST	4	1. VFSS
Guo and Zhang (41)	China	50 (28/22)	50 (33/17)	69.30 ± 12.18	67.00 ± 11.26	NMES + ST	ST	2	1. SSA 2. SWAL-QOL 3. WST 4. CR
Zheng et al. (42)	China	50 (26/24)	50 (25/25)	65.65 ± 15.53	65.16 ± 15.21	NMES + ST	ST	4	1. SSA 2. VFSS
Zhou et al. (43)	China	45 (25/20)	45 (23/22)	62.87 ± 3.57	63.18 ± 3.92	NMES + ST	ST	9	1. VFSS 2. WST
Zhu et al. (44)	China	20 (11/9)	20 (13/7)	56.6	56.1	NMES + ST	ST	2	1. VFSS
Dong (45)	China	50 (28/22)	50 (26/24)	48.25 ± 1.47	48.31 ± 1.44	NMES + ST	ST	3	1. SSA 2. SWAL-QO 3. WST
Liu (46)	China	28 (19/9)	28 (20/8)	58.9 ± 11.7	56.4 ± 10.3	NMES + ST	ST	8	1. UMHB 2. FMHB
Wu and Zhang (47)	China	20 (14/6)	20 (15/5)	57.6 ± 15.4	59.5 ± 17.6	NMES + ST	ST	3	1. WST
Xu et al. (48)	China	10 (6/4)	10 (4/6)	NA	NA	NMES + ST	ST	4	1. VFSS
Deng (49)	China	45 (25/20)	45 (24/21)	61.6 ± 4.9	61.8 ± 4.2	NMES + ST	ST	8	1. CR
Zhang (50)	China	52 (39/13)	52 (36/16)	65.90 ± 10.88	67.61 ± 10.44	NMES + ST	ST	2	1. CR
Li et al. (51)	China	45 (24/21)	45 (23/22)	66.7 ± 14.6	66.1 ± 13.1	NMES + ST	ST	4	1. SSA
Sproson et al. (52)	Britain	15 (10/5)	15 (9/6)	73 ± 15.3	81 ± 11.0	NMES + ST	ST	4	1. FOIS 2. PAS 3. SWAL-QOL
Simonelli et al. (53)	Italy	16 (10/6)	16 (6/10)	67.2 ± 16.2	72.4 ± 12.3	NMES + ST	ST	8	1. FOIS 2. PAS
Meng et al. (54)	China	10 (7/3)	10 (7/3)	65.2 ± 10.73	64.4 ± 9.03	NMES + ST	ST	2	1. WST 2. UMHB 3. FMHB
Park et al. (55)	South Korea	25 (12/13)	25 (14/11)	54 ± 11.93	55.8 ± 12.23	NMES + ST	Sham NMES + ST	6	1. PAS 2. FDS 3. UMHB 4. FMHB
Arreola et al. (56)	Spain	30 (19/11)	29 (19/10)	70.7 ± 12.91	73.52 ± 11.56	NMES + ST	ST	2	1. PAS
Carnaby et al. (57)	America	18 (10/8)	18 (8/10)	62.7 ± 12.2	70.6 ± 11.8	NMES + ST	Sham NMES + ST	3	1. CR
Huang et al. (58)	China	10 (9/1)	11 (6/5)	68.9 ± 16.9	67 ± 17.1	NMES + ST	ST	3	1. FOIS 2. PAS 3. FDS
Lee et al. (59)	South Korea	31 (22/9)	26 (20/6)	63.4 ± 11.4	66.7 ± 9.5	NMES + ST	ST	3	1. FOIS
Lim et al. (60)	South Korea	18 (12/6)	15 (10/5)	66.3 ± 15.4	62.5 ± 8.2	NMES + ST	ST	2	1. PAS 2. FDS
Zhang et al. (61)	China	27 (13/14)	28 (14/14)	63.72 ± 6.29	63.14 ± 6.56	NMES + ST	ST	6	1. CR
Guillén-Solà et al. (62)	Spain	21 (10/11)	21 (12/9)	70.3 ± 8.4	68.9 ± 7.0	NMES + ST	ST	3	1. CR
Zhang et al. (63)	China	28 (16/12)	27 (17/10)	61.3 ± 7.1	62.6 ± 8.7	NMES + ST	ST	4	1. FOIS 2. SSA 3. WST

NMES, transcutaneous neuromuscular electrical stimulation; ST, the swallowing rehabilitation training; T, treatment group; C, control group; NA, not available; SSA, the standardized swallowing assessment; SWAL-QOL, the swallowing quality of life questionnaire; WST, the water swallow test; VFSS, the videofluoroscopic swallow study; CR, complication rate; FMHB, forward movement distance of hyoid bone; UMHB, upward movement distance of hyoid bone; FOIS, functional oral intake scale; PAS, penetration-aspiration scale; FDS, functional dysphagia scale.

### 3.3. Bias risk evaluation of the included studies

**Figure 2:**
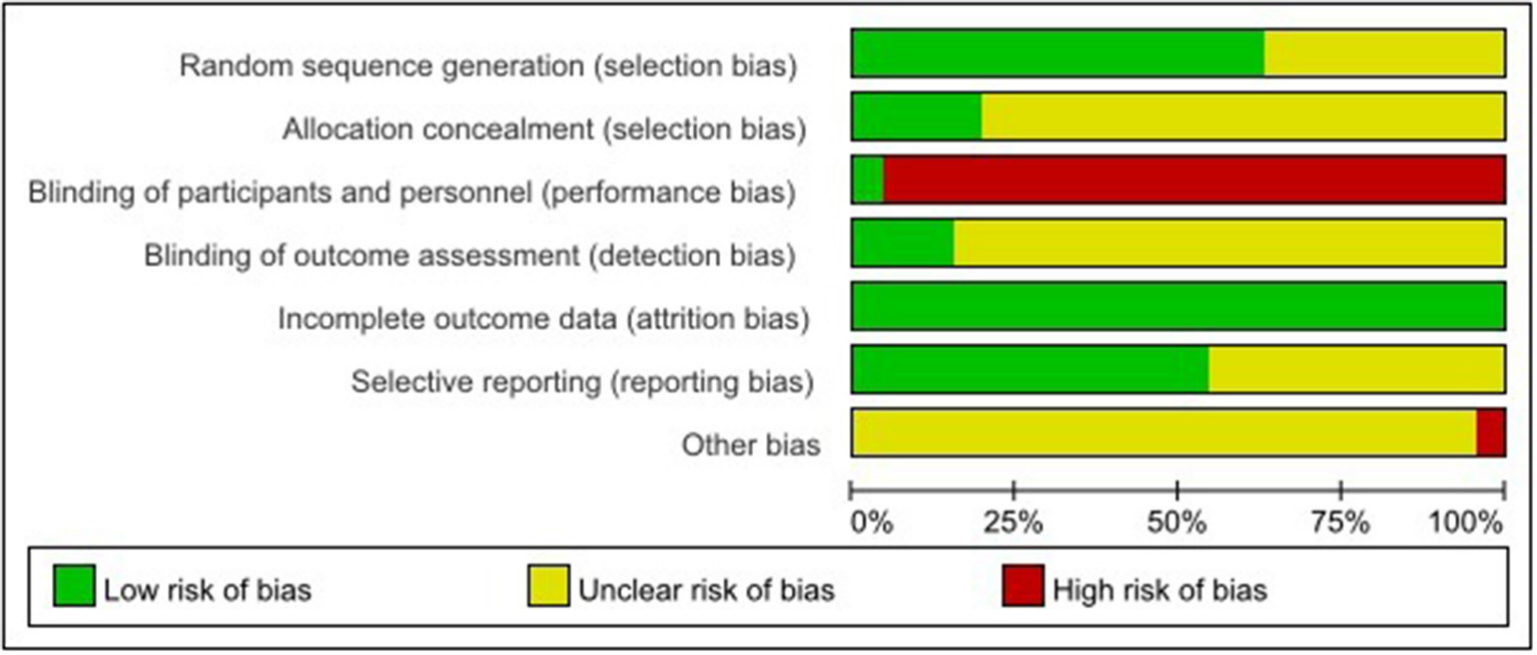
Risk of bias graph.

**Figure 3:**
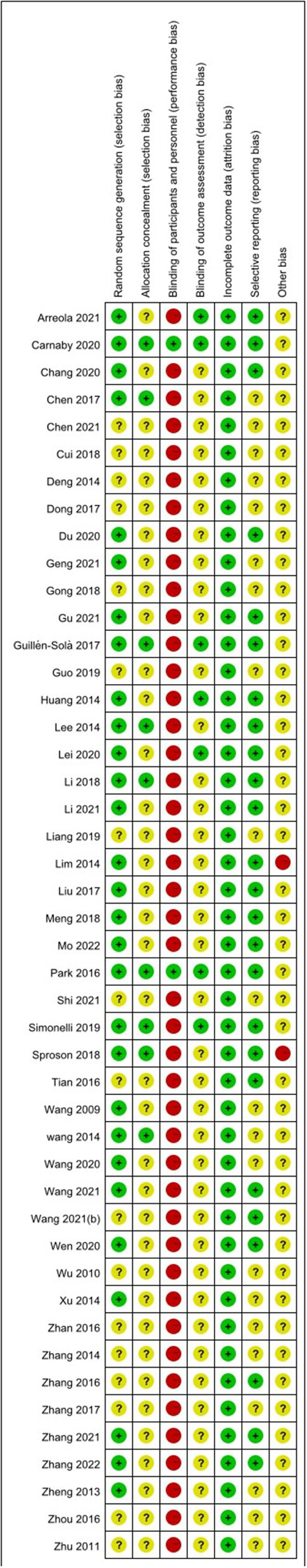
Risk of bias summary.

The risk of bias assessment is shown in Figures 2, 3.

#### 3.3.1. Generation of random sequence

Among the 46 studies included, 29 studies (18, 20, 22, 23, 25–27, 29, 32–34, 36, 37, 39, 42, 46, 48, 51–62) selected and reported appropriate randomization methods, such as random number table, so they were assessed as low risk of bias, the other 17 studies (19, 21, 24, 28, 30, 31, 35, 38, 40, 41, 43–45, 47, 49, 50, 63) only mentioned randomization allocation, so they were assessed as having the unclear risk of bias.

#### 3.3.2. Allocation concealment

Nine studies (20, 32, 51–53, 55, 57, 59, 62) followed the appropriate protocol to hide treatment allocation, so they were considered to have a low risk of bias. A total of 37 studies did not mention whether they followed the allocation hiding principle, so they were assessed as having an unclear risk of bias.

#### 3.3.3. Blinding of participants and personnel

Two studies (55, 57) explicitly proposed that the control group used sham NMES to blind participants and personnel, so they were assessed as having a low risk of bias because studies did not show that the control group received sham NMES. Instead, they only mentioned that the control group received ST and the experimental group received NMES + ST. We considered that participants were not blinded and rated studies as high risk of bias.

#### 3.3.4. Blinding of outcome assessment

Seven studies (26, 53, 55–58, 62) reported the blinding of outcome assessment, identifying it as a low risk of bias. The other 39 studies did not report whether to adopt the blinding of outcome assessment, identified as the unclear risk of bias.

#### 3.3.5. Incomplete outcome information

All studies thoroughly reported the test results data, so they were identified as having a low risk of bias.

#### 3.3.6. Selective reporting of study results

A total of 25 studies (18, 22, 25–27, 29, 31, 33, 35, 37, 39, 46, 51–63) reported the registration and ethical review of clinical RCTs, so the risk of bias was low. The remaining 21 studies were identified as having an unclear risk of bias.

#### 3.3.7. Other bias sources

Because there were high drop-out rates in two (52, 60) studies, we rated them as high risk. In the remaining 44 studies, we did not observe any potential study bias, so they were identified as the unclear risk of bias.

### 3.4. Results of the meta-analysis

#### 3.4.1. Standardized swallowing assessment

**Figure 4:**
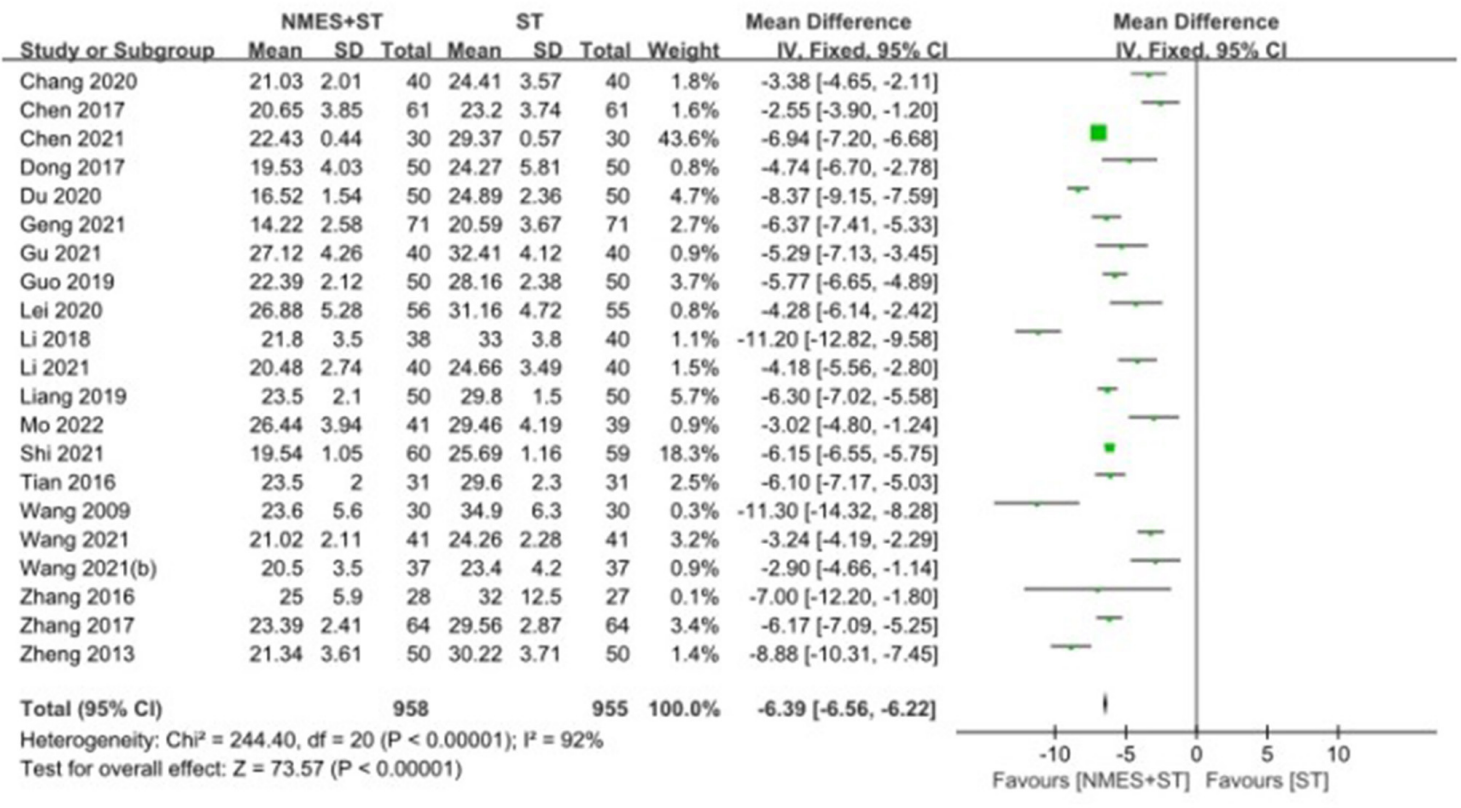
Forest plot for the standardized swallowing assessment.

A total of 21 studies reported changes in SSA in patients with post-stroke dysphagia after NMES + ST. The effect of NMES + ST on the improvement of swallowing function in patients with post-stroke dysphagia was better than single ST, and the difference was statistically significant [MD = −6.39, 95% CI (−6.56, −6.22), *P* < 0.00001, *I*^2^ = 92%], but there was heterogeneity as shown in Figure 4.

#### 3.4.2. Videofluoroscopic swallow study

**Figure 5:**
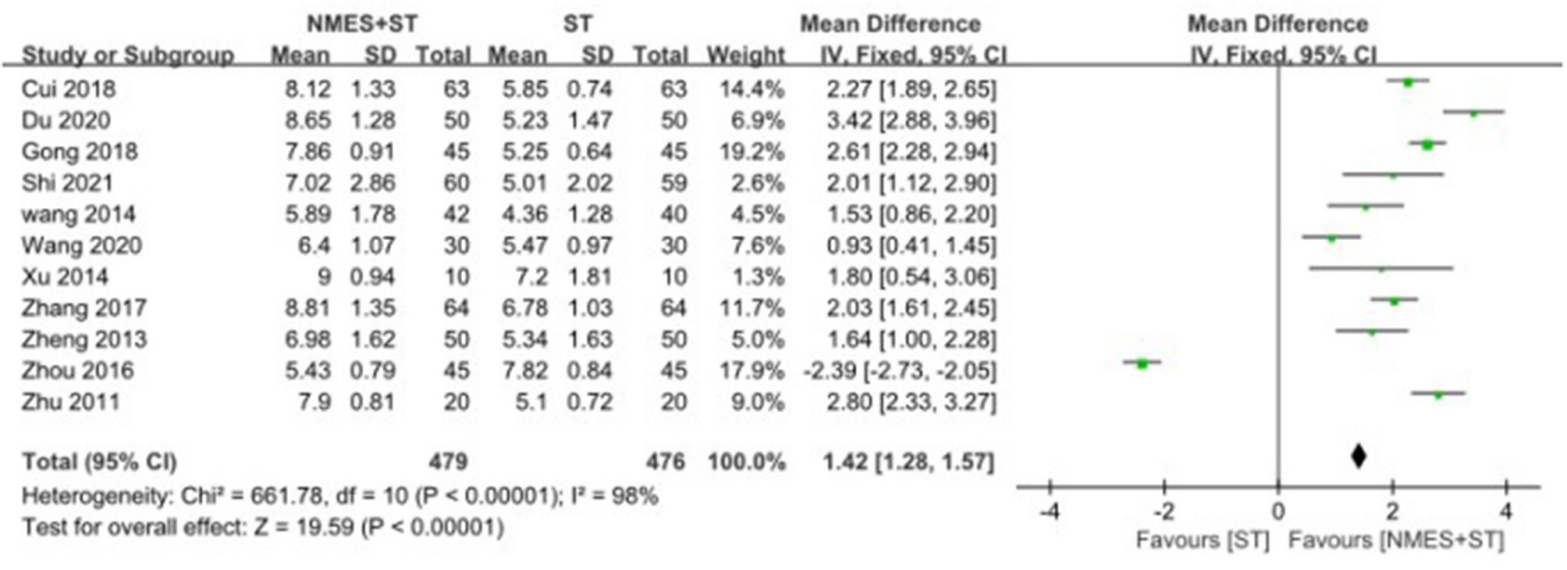
Forest plot for videofluoroscopic swallow study.

A total of 11 studies reported changes in VFSS after treatment, and NMES + ST was more effective [MD = 1.42, 95% CI (1.28, 1.57), *P* < 0.00001, *I*^2^ = 98%], but there was a high heterogeneity as shown in Figure 5.

#### 3.4.3. The swallowing quality of life questionnaire

**Figure 6:**
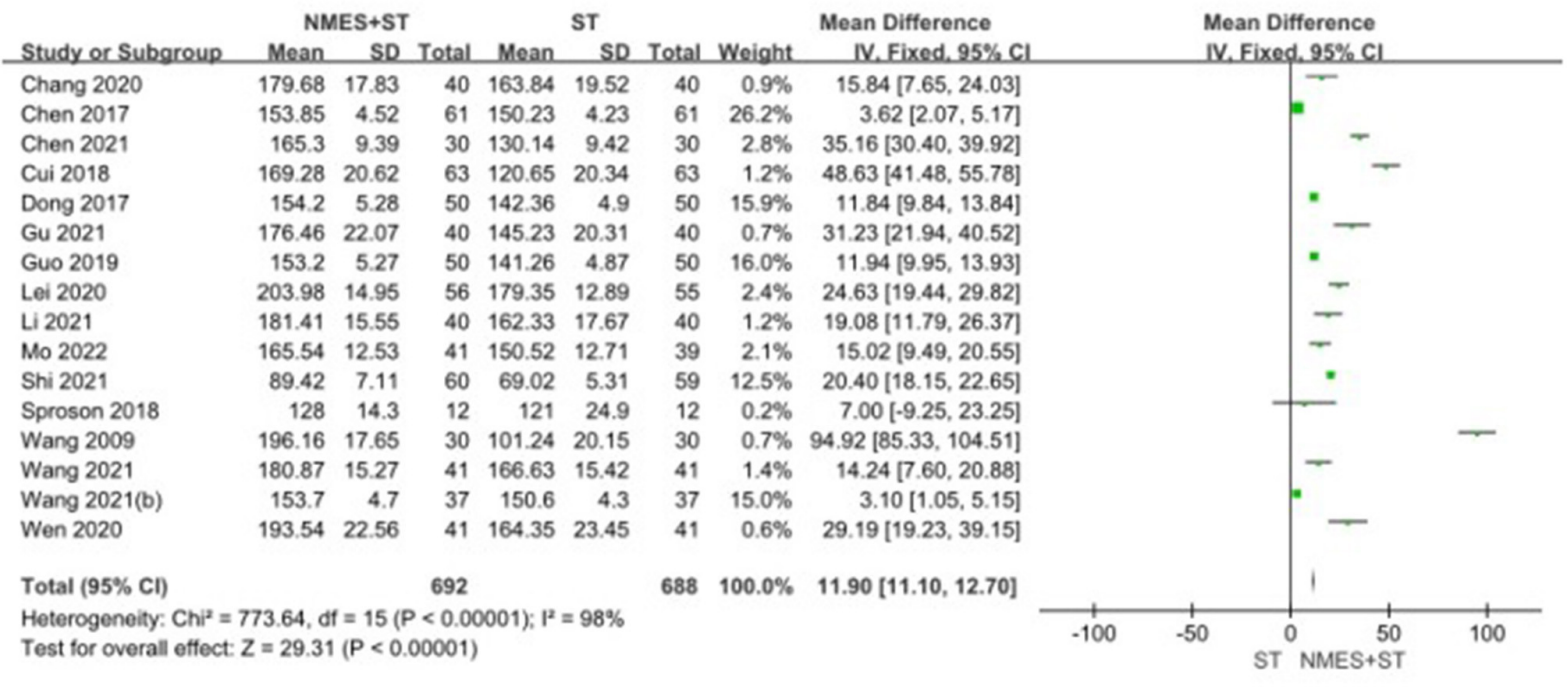
Forest plot for the swallowing quality of life questionnaire.

A total of 16 studies reported changes in SWAL-QOL scores after treatment. Compared with ST, NMES + ST improved the quality of life of patients with dysphagia more significantly [MD = 11.90, 95% CI (11.10, 12.70), *P* < 0.00001, *I*^2^ = 98%]. Nevertheless, there was heterogeneity as shown in Figure 6.

#### 3.4.4. Water swallow test

**Figure 7:**
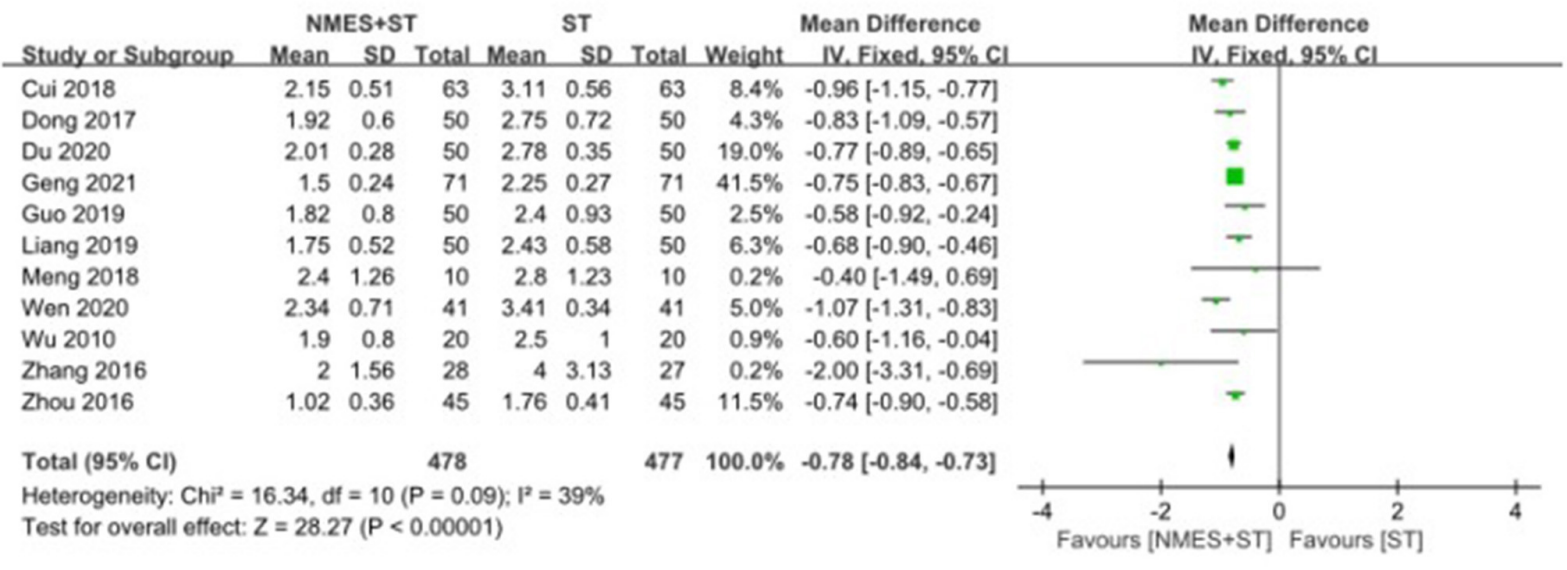
Forest plot for water swallow test.

A total of 11 studies used the water swallow test to evaluate the swallowing function of patients. The efficacy of NMES + ST was better than that of single ST, with the statistical difference [MD = −0.78, 95% CI (−0.84, −0.73), *P* < 0.00001, *I*^2^ = 39%], as shown in Figure 7.

#### 3.4.5. Forward movement distance of the hyoid bone

**Figure 8:**
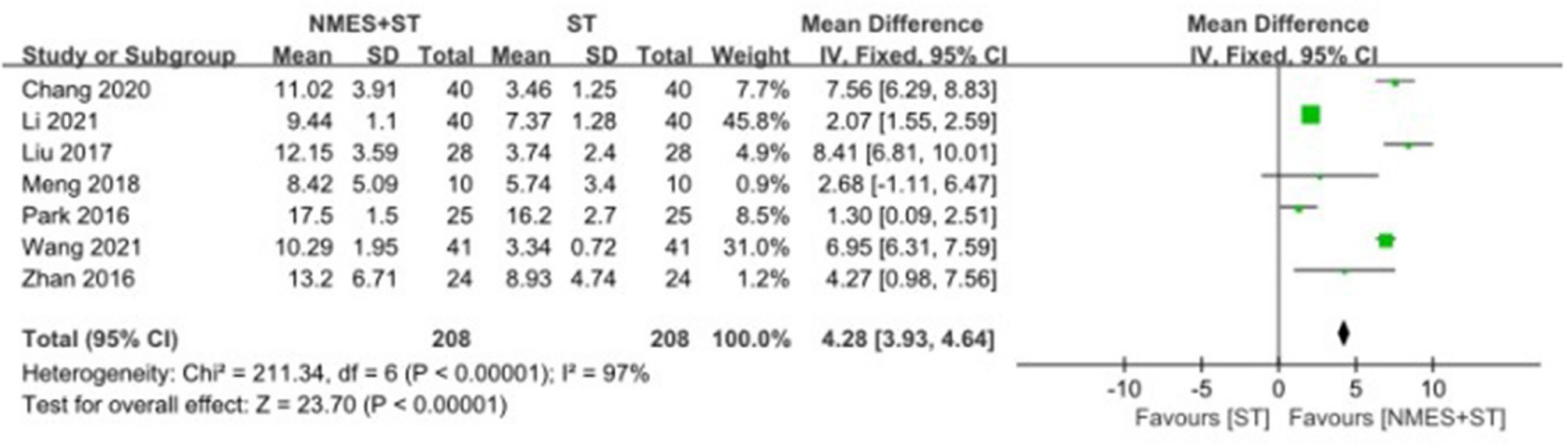
Forest plot for forward movement distance of hyoid bone.

Seven studies reported changes in the forward movement distance of the hyoid bone after treatment. NMES + ST increased the forward movement distance of hyoid bone compared with single ST [MD = 4.28, 95% CI (3.93, 4.64), *P* < 0.00001, *I*^2^ = 97%], but there was heterogeneity, as shown in Figure 8.

#### 3.4.6. Upward movement distance of the hyoid bone

**Figure 9:**
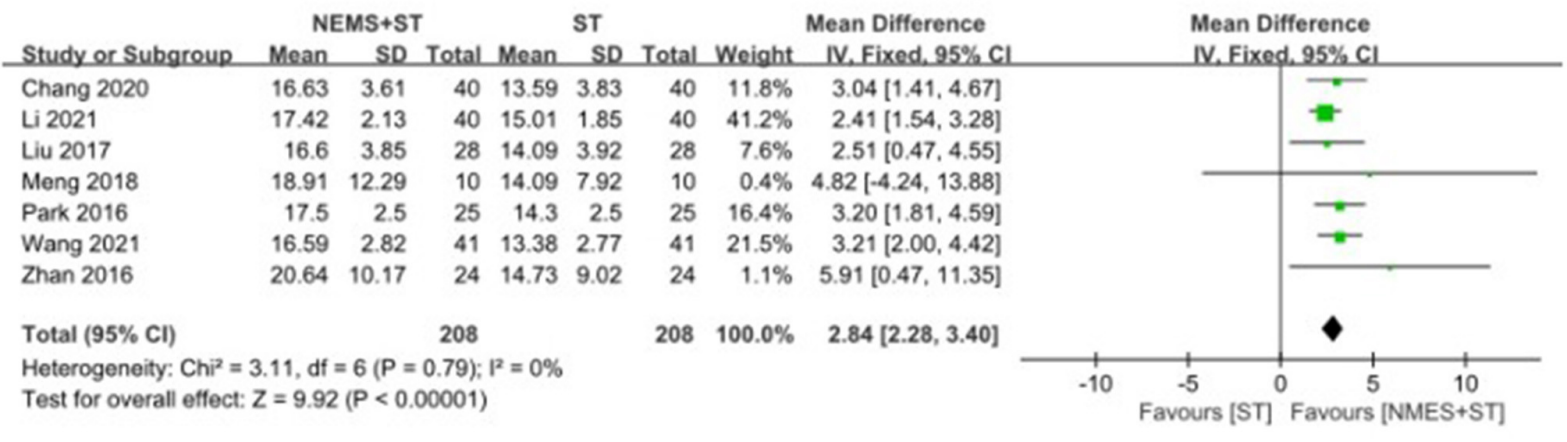
Forest plot for upward movement distance of hyoid bone.

Seven studies reported changes in the upward movement distance of the hyoid bone after treatment. NMES + ST significantly increased the upward movement distance of the hyoid bone compared with ST [MD = 2.84, 95% CI (2.28, 3.40), *P* < 0.00001, *I*^2^ = 0%], as shown in Figure 9.

#### 3.4.7. Complication rate

**Figure 10:**
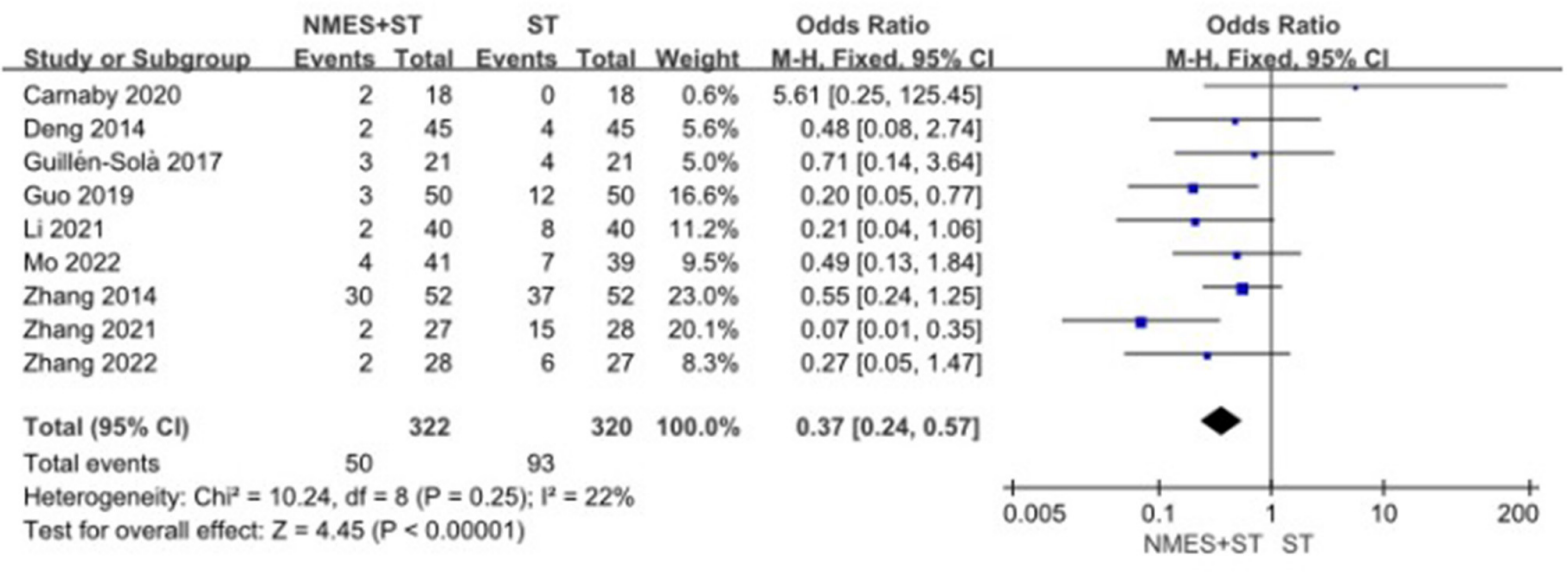
Forest plot for complication rate.

Nine studies evaluated and recorded the rate of complications in patients with dysphagia. The results showed that NMES + ST could significantly reduce the rate of complications such as pneumonia and malnutrition compared with single ST (OR = 0.37 95% CI [0.24, 0.57], *P* < 0.00001, *I*^2^ = 22%), as shown in Figure 10.

#### 3.4.8. Penetration-aspiration scale

Six studies reported changes in PAS after treatment, with NMES + ST achieving better clinical efficacy than ST [MD = −0.63, 95% CI (−1.15, −0.12), *P* = 0.01, *I*^2^ = 14%; as shown in the Supplementary material].

#### 3.4.9. Functional oral intake scale

Five studies reported changes in FOIS after treatment, and NMES + ST was better than ST [MD = 1.32, 95% CI (0.81, 1.83), *P* < 0.00001, *I*^2^ = 18%; as shown in the Supplementary material].

#### 3.4.10. Functional dysphagia scale

Three studies evaluated swallowing function according to FDS, and the clinical effect of NMES + ST may be more positive [MD = −8.81, 95% CI (−16.48, −1.15), *P* = 0.02, *I*^2^ = 57%; as shown in the Supplementary material].

### 3.5. Subgroup analysis

#### 3.5.1. Subgroup analysis of SSA

A subgroup analysis showed that 25 Hz electrical stimulation [MD = −7.00, 95% CI (−12.20, −1.80), *P* = 0.008] had a more positive clinical effect on dysphagia after stroke than 10–50 Hz electrical stimulation [MD = −6.17, 95% CI (−7.09, −5.25), *P* < 0.00001], 30–80 Hz electrical stimulation [MD = −5.62, 95% CI (−8.18, −3.06), *P* < 0.0001], 40–80 Hz electrical stimulation [MD = −3.02, 95% CI (−4.80, −1.24), *P* = 0.0009], 80 Hz electrical stimulation [MD = −5.13, 95% CI (−7.68, −2.59), *P* < 0.0001]. In the study, 7 mA electrical stimulation [MD = −11.20, 95% CI (−12.82, −9.58), *P* < 0.00001] was better than 0–25 mA [MD = −5.83, 95% CI (−7.48, −4.19), *P* < 0.00001], 0–15 mA [MD = −8.08, 95% CI (−11.80, −4.37), *P* < 0.0001], 5–11 mA [MD = −3.85, 95% CI (−6.53, −1.16), *P* = 0.005], 5–25 mA [MD = −6.37, 95% CI (−6.99, −5.75), *P* < 0.00001], 0–30 mA [MD = −4.28, 95% CI (−6.14, −2.42), *P* < 0.00001]. A 4-week treatment course [MD = −6.29, 95% CI (−7.16, −5.42), *P* < 0.00001] might have better clinical efficacy, and older patients (age > 60 years old; MD = −6.33, 95% CI [−7.10, −5.56], *P* < 0.00001) may have a more significant positive effect on post-stroke dysphagia than younger patients (age <60 years old; MD = −4.58, 95% CI [−6.03, −3.14], *P* < 0.00001). However, in the duration of each treatment and course of the disease, there is no statistical difference between subgroups (as shown in the Supplementary material).

#### 3.5.2. Subgroup analysis of VFSS

The subgroup analysis showed that the treatment group within 4 weeks [MD = 2.24, 95% CI (1.62, 2.86), *P* < 0.00001] was better than the 4-week treatment group [MD = 2.09, 95% CI (1.46, 2.71), *P* < 0.00001] and the treatment group over 4 weeks [MD = −2.39, 95% CI (−2.73, −2.05), *P* < 0.00001]. There was no significant difference in clinical efficacy between subgroups in age, course of the disease, duration of each treatment, intensity of electrical stimulation, and frequency of electrical stimulation (as shown in the Supplementary material).

#### 3.5.3. Subgroup analysis of SWAL-QOL

Subgroup analysis showed that 0–15 mA electrical stimulation [MD = 94.92, 95% CI (85.33, 104.51), *P* < 0.00001] was compared with 0–25mA [MD = 21.00, 95% CI (10.04, 31.96), *P* = 0.0002], 5–11 mA [MD = 27.68, 95% CI (−3.73, 59.09), *P* = 0.08], 0–30 mA [MD = 24.63, 95% CI (19.44, 29.82), *P* < 0.00001], 14–20 mA [MD = 29.19, 95% CI (19.23, 39.15), *P* < 0.00001] might produce better influence. Patients [the day from onset <20 days; MD = 30.32, 95% CI (12.27, 48.37), *P* = 0.001] may have better clinical efficacy, and older patients (age > 60 years old; MD = 27.50, 95% CI [18.58, 36.42], *P* < 0.00001) was better than younger patients (age <60 years old; MD = 13.25, 95% CI [5.67, 20.84], *P* = 0.0006). However, there were no statistical differences between subgroups in electrical stimulation frequency, course of treatment, and duration of each treatment (as shown in the Supplementary material).

### 3.6. Publication bias

**Figure 11:**
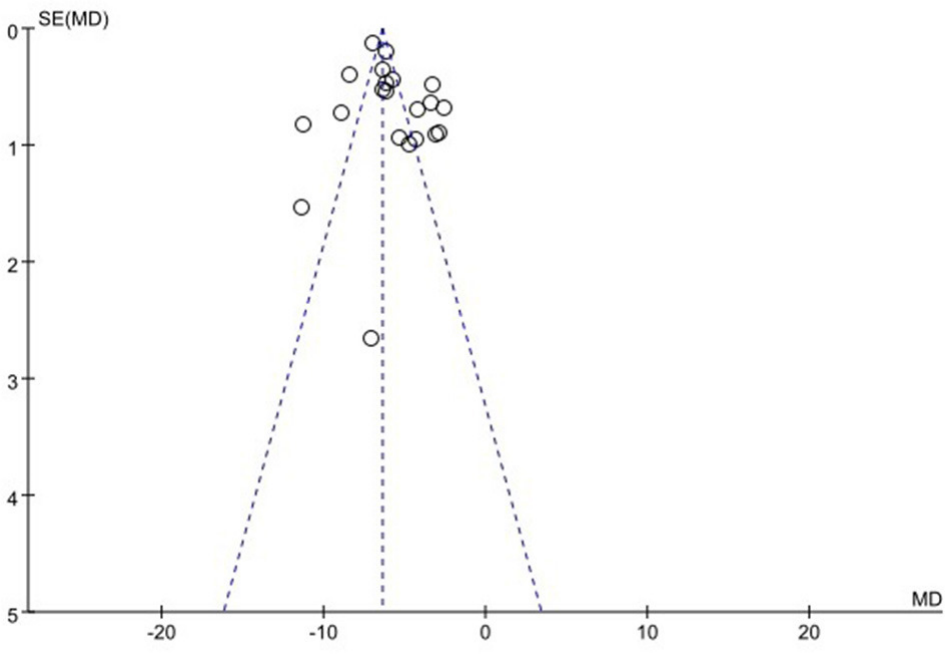
Funnel plot for standardized swallowing assessment.

**Figure 12:**
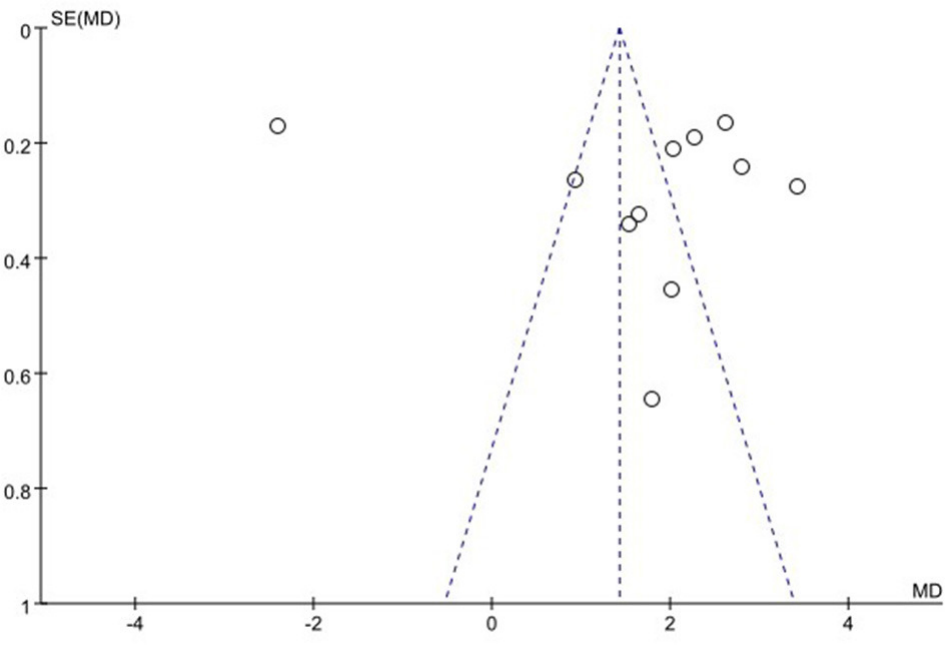
Funnel plot for videofluoroscopic swallow study.

**Figure 13:**
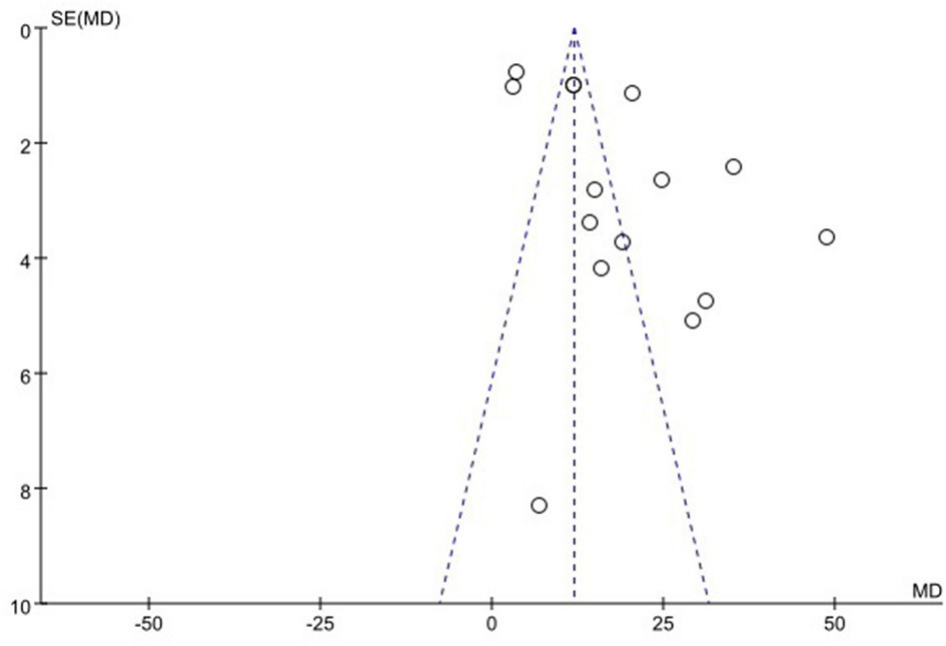
Funnel plot for the swallowing quality of life questionnaire.

We used funnel plots to evaluate the publication bias of SSA, VFSS, SWAL-QOL, and WST, respectively. The results showed that the funnel plots of SSA and WST were relatively symmetric, and they might not have publication bias, while the funnel plots of VFSS and SWAL-QOL were asymmetric. It might have publication bias, as shown in Figures 11–13, Supplementary material.

### 3.7. Sensitivity analysis

Sensitivity analysis was performed because *I*^2^ of SSA (92%), VFSS (98%), SWAL-QOL (98%), FMHB (97%), and FDS (57%) were >50%. After sensitivity analysis of FDS, we excluded one study that could have led to high heterogeneity. FDS analysis showed the disappearance of high heterogeneity (*I*^2^ = 0; Supplementary material). However, after sensitivity analysis of other relevant literature data, the responsible articles leading to high heterogeneity were not determined. During the analysis, we found that some subgroups still had high heterogeneity. Subsequently, sensitivity analysis was conducted again for each subgroup, but the source of heterogeneity was still not identified. Therefore, meta-regression analysis was used to explain the high heterogeneity. However, it was not performed due to the small number of studies included in FMHB (as shown in the Supplementary material).

#### 3.7.1. Heterogeneity of SSA

According to the results of the meta-regression analysis, the high heterogeneity of SSA may be related to age (*P* = 0.032), but there was not significantly associated with the course of treatment (*P* = 0.160), course of disease (*P* = 0.091), duration of each treatment (*P* = 0.096), the intensity of electrical stimulation (*P* = 0.320), and frequency of electrical stimulation (*P* = 0.802).

#### 3.7.2. Heterogeneity of SWAL-QOL

The heterogeneity of SWAL-QOL results might be due to the age (*P* = 0.024), course of treatment (*P* = 0.003), course of disease (*P* = 0.002), and duration of each treatment (*P* = 0.003), but there was no significant correlation with frequency of electrical stimulation (*P* = 0.782) and intensity of electrical stimulation (*P* = 0.287).

#### 3.7.3. Heterogeneity of VFSS

The heterogeneity of VFSS results might be due to the course of treatment (*P* = 0.022), but it might not be related to age (*P* = 0.147), course of disease (*P* = 0.345), treatment time (*P* = 0.124), the intensity of electrical stimulation (*P* = 0.459), and frequency of electrical stimulation (*P* = 0.542).

### 3.8. Quality of evidence

After the GRADE evaluation, the quality of evidence: WST and UMHB were rated as moderate quality. VFSS, CR, FMHB, and FOIS were rated as low quality. PAS, FDS, SSA, and SWAL-QOL were rated as very low quality. The low quality included the high risk of bias in the included studies, insufficient sample size, high heterogeneity, indirect comparison of trial results, and inconsistency as shown in Table 2.

**Table 2:** Summary of GRADE recommendations.

**Quality assessment**								**No. of patients**		**Effect**		**Quality**
**Outcome**	**No. of studies**	**Study design**	**Risk of bias**	**Inconsistency**	**Indirectness**	**Imprecision**	**Other considerations**	**NMES** + **ST**	**ST**	**Relative (95% CI)**	**Absolute**	
Standardized swallowing assessment	21	Randomized trials	Serious^1^	Serious^2^	Serious^3^	None	None	958	955		MD 6.39 lower (6.56–6.22 lower)	Very low
The swallowing quality of life questionnaire	16	Randomized trials	Serious^1^	Serious^2^	Serious^3^	None	None	692	688		MD 11.90 higher (11.10–12.70 higher)	very low
Penetration-aspiration scale	6	Randomized trials	Serious^1^	Serious^4^	None	Serious^4^	None	111	107		MD 0.63 lower (1.15–0.12 lower)	Very low
Water swallow test	11	Randomized trials	Serious^1^	None	None	None	None	478	477		MD 0.78 lower (0.84–0.73 lower)	Moderate
Functional dysphagia scale	3	Randomized trials	Serious^1^	Serious^2^	None	Serious^4^	None	53	51		MD 8.81 lower (16.48–1.15 lower)	Very low
Functional Oral Intake Scale	5	Randomized trials	Serious^1^	None	None	Serious^4^	None	97	93		MD 1.32 higher (0.81–1.83 higher)	Low
Videofluoroscopic Swallow Study	11	Randomized trials	Serious^1^	Serious^2^	None	None	None	479	476		MD 1.42 higher (1.28–1.57 higher)	Low
Forward movement distance of hyoid bone	7	Randomized trials	Serious^1^	Serious^2^	None	None	None	208	208		MD 4.28 higher (3.93–4.64 higher)	Low
Upward movement distance of hyoid bone	7	Randomized trials	Serious^1^	None	None	None	None	208	208		MD 2.84 higher (2.28–3.40 higher)	Moderate
**Quality assessment**								**No. of patients**		**Effect**		**Quality**
**Outcome**	**No. of studies**	**Study design**	**Risk of bias**	**Inconsistency**	**Indirectness**	**Imprecision**	**Other considerations**	**NMES** + **ST**	**ST**	**Relative (95% CI)**	**Absolute**	
Complication rate	9	Randomized trials	Serious^1^	Serious^4^	None	None	None	50/322 (15.5%)	93/320 (29.1%)	OR 0.37 (0.24 to 0.57)	159 fewer per 1,000 (from 101 to 201 fewer)	Low
									20%		115 fewer per 1,000 (from 75 to 143 fewer)	

Supplement: 1. High risk of bias; 2. High heterogeneity; 3. Indirect comparison; 4. The number of the patient included is small; 4. Inconsistent results.

### 3.9. Adverse events

Nine studies reported adverse events. Five studies (50–53, 62) reported that during the study period, patients in the experimental group did not report any adverse events, while Wang et al. (33) reported that one patient had a skin allergy to the electrode patch; one patient had a peculiar smell in the mouth, and the adverse reactions disappeared after stopping treatment. Arreola (56) and Lim (60) show that two patients reported skin tingling, which disappeared after electrical stimulation was stopped. Zhang et al. (63) reported that seven patients had localized skin redness or allergic reactions in the area of electrode placement, and the adverse reactions disappeared after the treatment was stopped, and no one withdrew because of skin reactions.

## 4. Discussion

This meta-analysis showed that NMES + ST could effectively increase the forward and upward movement distance of the hyoid bone, improve the quality of life of patients with dysphagia, reduce the rate of complications, and improve the swallowing function of patients.

In addition, subgroup analysis based on the course of the disease found that NMES + ST may have a more significant clinical influence on patients (the day from onset <20 days). This subgroup results emphasize the importance of early treatment of dysphagia after stroke, which may be related to the fact that NMES can enhance pharyngeal sensory feedback pathways, promote cortical reorganization, and increase pharyngeal motor performance in the contralateral motor cortex (64–66). Early treatment may be more effective in promoting cortical reorganization.

The subgroup analysis based on the course of treatment showed that 4 weeks or less might achieve more satisfactory clinical efficacy than the course of more than 4 weeks. The study (67) found that electrical stimulation may have a cumulative effect on brain activity, which is associated with the recovery of behavioral function. Therefore, we wondered if there was an upper threshold for the cumulative effect of electrical stimulation on the plasticity of the cerebral cortex, resulting in the limited recovery of swallowing function from excessive electrical stimulation.

In the subgroup analysis based on the frequency and intensity of electrical stimulation, the results of SSA as the outcome index showed that 25 Hz electrical stimulation and 7 mA electrical stimulation seemed to have a more positive effect. In contrast, the results of SWAL-QOL showed that the clinical efficacy of 0–15 mA electrical stimulation was more prominent. However, there was no statistical difference between the subgroups of electrical stimulation at different frequencies (*P* > 0.05). Studies (63, 68) confirmed that there were specific differences in clinical efficacy in different intensities and frequencies of electrical stimulation, which might be related to the different degrees of motor-evoked potentials (MEPs) induced by different frequencies and intensities of stimulation on the pharyngeal muscle. Pharyngeal muscle MEPs were closely related to the excitability of the swallowing cortex-medulla oblongata (69, 70) and could have a long-term effect on the reorganization of the cerebral cortex through nerve conduction, thereby promoting the recovery of swallowing function.

In age-based subgroup analysis, it appeared to be more clinically positive in patients (age > 60 years old) than in patients (age <60 years old). As people grow older, the human physiological function will also decrease (71), which may lead to the fact that routine ST does not provide the same recovery effect for older patients as for younger patients. In the control group, patients (age > 60 years old) achieved worse clinical efficacy in conventional ST than patients (age <60 years old). However, patients in the experimental group received NMES + ST. The addition of electrical stimulation can enhance the contraction of swallowing muscles, promote the repair of damaged nerves and the remodeling of the cerebral cortex, which may make it possible that patients (age > 60 years old) in the experimental group can obtain the same benefits as other patients (age <60 years old). For this reason, the relative benefit is more significant in patients over 60 than in patients under 60.

Swallowing is a complex neuromuscular reflex activity involving the cortical center, brainstem swallowing center, peripheral nerve, and other aspects. Stroke can cause damage to the cortical swallowing center, corticobulbar tract, brainstem swallowing center, cranial nerves (V, IX, X, XI, and XII), and spinal nerves (C1, C2, and C3), which leads to symptoms of swallowing disorder such as drinking cough, eating difficulties, dysarthria. Research (72, 73) has proved that specific intensity of electrical stimulation on the glossopharyngeal muscle group can enhance muscle contraction ability, increase the degree of activation, and prevent disuse muscle atrophy. Swallowing-related muscles are mainly composed of type I muscle fibers and type II muscle fibers. Type II muscle fibers are smaller than type I and are not easily polarized (73). When NMES stimulates muscle, type II muscle fibers that constitute swallowing muscles are preferentially activated. Traditional rehabilitation training activates type I muscle fibers (72). When simultaneous treatment is performed, type I and type II muscle fibers are simultaneously activated, and the glossopharyngeal muscle group can produce a stronger contraction. In addition, electrical stimulation can produce vasoactive peptides to cause local vasodilation, which can improve the blood circulation of the injured part (74), accelerate the regeneration and repair of the nerve to correctly project the regeneration track of the target organ, promote the regeneration of the axon and the maturation of the myelin sheath (75), and further promote the functional recovery and reorganization of the cerebral cortex and related neural connections and pathways (76, 77). The sufficient movement of the hyoid-laryngeal complex is the key to ensuring the effective and safe completion of swallowing activities (78, 79). At present, the range of motion of the hyoid bone is often used to measure the movement of the hyoid-laryngeal complex of patients with dysphagia. Furthermore, the upward and forward movement distance of the hyoid bone in patients with dysphagia is significantly lower than that of ordinary people (80–82). This study also shows that while the swallowing function of patients is improved, the distance of upward and forward movement of the hyoid bone is significantly increased, which is consistent with previous studies.

However, in the subgroup analysis of the frequency and intensity of electrical stimulation, there was high heterogeneity within subgroups possibly due to the different placement of NMES in different studies. Studies had found that when electrodes were placed on the suprahyoid muscle (83), thyrohyoid muscle (53), orbicularis oculi muscle (84), and masseter muscle (85), the swallowing functions were improved. Furthermore, a meta-analysis study showed that horizontal electrodes placed in the suprahyoid muscle or suprahyoid muscle and thyrohyoid muscle seem to have the best effect (11), so the electrode placement site is related to clinical efficacy. The heterogeneity of the treatment course subgroup may be related to the differences in the intensity and frequency of electrical stimulation, pulse duration, and swallowing rehabilitation. One study (86) proposed that the shorter the pulse duration, the greater the stimulation intensity needed to obtain a muscle response. The pulse duration is inversely proportional to the specificity of the stimulus applied, which may be responsible for the high heterogeneity in subgroups. In addition, the high heterogeneity of age and disease course subgroups may be related to the inconsistency of stroke type, disease severity, frequency of electrical stimulation, and electrical stimulation intensity among patients included in different studies, which still needs further exploration.

### 4.1. Strengths and weaknesses

This study included more clinical randomized controlled trials (46 RCTs) and case numbers (3,346 patients) than previous meta-analysis studies on NMES in treating post-stroke dysphagia. It evaluated the clinical efficacy of NMES + ST from 10 different outcome indicators, such as SSA, SWAL-QOL, and VFSS. In addition, subgroup analysis found that the clinical efficacy of NMES + ST in patients with a disease course of fewer than 20 days appeared to be more significant than that in patients with a disease course of more than 20 days, which provided evidence support for early intervention treatment. A treatment course of 4 weeks or less appears to be better than a course of more than 4 weeks, which will help reduce the cost of treatment and improve the potential cost-effectiveness of the intervention; Electrical stimulation with a frequency of 25 Hz and intensity of 7 mA or 0–15 mA appears to work better. This finding could help develop optimal stimulation parameters. The effect of electrical stimulation in patients over 60 years old is noticeable, promoting further attention to the treatment of swallowing disorders in the elderly. This study also has some limitations: (1) The majority of the 46 RCTs included in this study are from China, which may lead to regional bias. (2) Most of the included clinical trials do not report the blinding method used, which reduced the quality of the methodological study. There may be some placebo effect and observer bias, which may reduce the credibility of the clinical trial results. (3) Among 46 RCTs, only six studies followed-up visited with the patients, so the long-term clinical efficacy of NMES + ST on post-stroke dysphagia still needs to be further explored. (4) Adverse events were reported in only some studies, which resulted in insufficient evidence to support the safety of NMES + ST treatment. Future studies still need to strengthen the recording and reporting of adverse events. (5) Due to the high heterogeneity, some results' reliability in the study has been somewhat affected. (6) The use of multiple types of ST in different studies led to the fact that this study did not perform a subgroup analysis based on the type of ST received by the control group. We look forward to further exploration in subsequent studies.

## 5. Conclusion

Our study showed that NMES + ST could effectively increase the forward and upward movement distance of the hyoid bone, improve the quality of life of patients with post-stroke dysphagia, reduce the rate of complications, and promote the recovery of swallowing function. NMES with a frequency of 25 Hz, an intensity of 0–15 mA, and a treatment course of 4 weeks or less may have better results. Patients with an onset of fewer than 20 days and over 60 years old appear more effective with NMES + ST. However, there is insufficient evidence on the safety of NMES + ST for post-stroke dysphagia. Moreover, due to the small number of included literature and the low quality of evidence, more large-sample, high-quality, multi-center RCT studies are needed to prove the clinical efficacy of NMES + ST in the treatment of post-stroke dysphagia.

## Data availability statement

The original contributions presented in the study are included in the article/Supplementary material, further inquiries can be directed to the corresponding author.

## Author contributions

YW conceived the study and drafted the protocol. YW, LX, and LZ participated in literature screening, data extraction, and bias risk assessment. YW, LW, MJ, and LX are responsible for statistical analysis and manuscript writing. LZ was responsible for the planning and guidance of this article. All authors participated in the study and approved the published version of the manuscript.
