# Supplementary material for: Effects of transcutaneous neuromuscular electrical stimulation on post-stroke dysphagia: a systematic review and meta-analysis
Source: Front Neurol. 2023 May 9;14:1163045. doi: 10.3389/fneur.2023.1163045 (PMC10203701; doi:10.3389/fneur.2023.1163045)
Supplement: Supplementary file 1 [file Data_Sheet_1.docx]

Supplementary Material

**Effects of transcutaneous neuromuscular electrical stimulation on post-stroke dysphagia： a systematic review and meta-analysis**

# Supplementary Tables

Supplementary table1. The search strategy

| **NO.** | **Search Items** |
| --- | --- |
| **#1** | Transcutaneous Electric Stimulation OR Percutaneous Electric Nerve Stimulation OR Transcutaneous Electrical Stimulation OR Transdermal Electrostimulation OR Percutaneous Electrical Nerve Stimulation OR Transcutaneous Electrical Nerve Stimulation OR Transcutaneous Nerve Stimulation OR TENS OR Percutaneous Neuromodulation Therapy OR Percutaneous Electrical Neuromodulation OR Percutaneous Electrical Neuromodulations OR Analgesic Cutaneous Electrostimulation OR PENS [Title/Abstract] |
| **#2** | Stroke OR Cerebrovascular Accident OR CVA (Cerebrovascular Accident) OR Cerebrovascular Apoplexy OR Brain Vascular Accident OR Cerebrovascular Stroke OR Apoplexy OR Cerebral Stroke OR Acute Stroke OR Acute Cerebrovascular Accident [Title/Abstract] |
| **#3** | Deglutition Disorder OR Deglutition Dysfunction OR Swallowing Disorder OR Swallowing Dysfunction OR Dysphagia OR Oropharyngeal Dysphagia OR Esophageal Dysphagia [Title/Abstract] |
| **#4** | Randomized controlled trial OR Controlled clinical trial [Publication Type] |
| **#5** | Randomized OR Random OR Randomly OR trial OR RCT [Title/Abstract] |
| **#6** | #4 OR #5 |
| **#7** | #1 AND #2 AND #3 AND #6 |

Supplementary Table 2 Subgroup analysis data extraction table

| study | Age(y) | Course of disease(d) | Course of treatment（W） | Time of therapy(min) | Current intensity（mA） | Current frequency(Hz) |
| --- | --- | --- | --- | --- | --- | --- |
| Chang2020^[19]^ | ＞60 | ＜20 | ＞4 | 20 | NA | NA |
| Chen2021^[20]^ | ＞60 | ＜20 | 4 | 20 | 0~25 | NA |
| Chen2017^[21]^ | ＜60 | ＞20 | 4 | 20 | 5~11 | 30~80 |
| Cui2018^[22]^ | ＞60 | ＞20 | ＜4 | 20 | 5~11 | 30~80 |
| Du2020^[23]^ | ＞60 | NA | 4 | 25 | 0~25 | 30~80 |
| Geng2021^[24]^ | ＜60 | ＞20 | 4 | 20 | 5~25 | NA |
| Gong2018^[25]^ | ＜60 | NA | 4 | NA | 0~25 | 80 |
| Gu2021^[26]^ | ＞60 | ＞20 | ＞4 | 20 | 5~11 | 30~80 |
| Lei2020^[27]^ | ＜60 | ＞20 | ＜4 | 20 | 0~30 | 80 |
| Li2021^[28]^ | ＞60 | ＞20 | ＞4 | 30 | 0~25 | 80 |
| Liang2019^[29]^ | ＞60 | ＞20 | ＜4 | 30 | NA | NA |
| Mo2022^[30]^ | ＞60 | ＜20 | 4 | 60 | 0~25 | 40~80 |
| Shi2021^[31]^ | ＞60 | ＞20 | 4 | 20 | NA | NA |
| Tian2016^[32]^ | ＜60 | ＞20 | 4 | 30 | 0~15 | 30~80 |
| Wang2014^[33]^ | NA | NA | ＜4 | 30 | 0~25 | NA |
| Wang2021^[34]^ | ＜60 | ＜20 | 4 | 20 | 0~25 | 80 |
| Wang2020^[35]^ | ＞60 | NA | 4 | 20 | 0~25 | 80 |
| Wang2021B^[36]^ | ＞60 | NA | 4 | 30 | NA | NA |
| Wang2009^[37]^ | ＞60 | ＜20 | 4 | 60 | 0~15 | NA |
| Wen2020^[38]^ | ＞60 | ＜20 | 4 | 30 | 14~20 | 80 |
| Zhang2017^[41]^ | ＞60 | NA | 4 | 20 | NA | 10~50 |
| Guo2019^[42]^ | ＞60 | NA | ＜4 | 20 | NA | NA |
| Zheng2013^[43]^ | ＞60 | ＞20 | 4 | 30 | 0~25 | 80 |
| Zhou2016^[44]^ | ＞60 | ＜20 | ＞4 | 30 | 5~11 | 30~80 |
| Zhu2011^[45]^ | ＜60 | ＜20 | ＜4 | 60 | 5~11 | 30~80 |
| Dong2017^[46]^ | ＜60 | ＞20 | ＜4 | 20 | NA | NA |
| Xu2014^[49]^ | NA | NA | 4 | 60 | NA | NA |
| Li2018^[52]^ | ＞60 | ＜20 | 4 | 60 | 7 | NA |
| Sproson2018[53] | ＞60 | ＜20 | 4 | 30 | NA | 30 |
| Simonelli2019[54] | ＞60 | ＞20 | 8 | 30 | 7.8-12.5 | 80 |
| Meng2018[55] | ＞60 | ＞20 | 2 | 30 | 0-25 | 80 |
| Park2016[56] | ＜60 | ＞20 | 6 | 30 | 9-14 | 80 |
| Arreola2021[57] | ＞60 | ＞20 | 2 | 60 | 15.14±5.72 | 80 |
| Carnaby2020[58] | ＞60 | ＜20 | 3 | 60 | 6.63±3.2 | NA |
| Huang2014[59] | ＞60 | ＞20 | 3 | 60 | 0-25 | 80 |
| Lee2014[60] | ＞60 | ＜20 | 3 | 30 | NA | 80 |
| Lim2014[61] | ＞60 | ＞20 | 2 | 30 | 7-9 | 80 |
| Zhang2021[62] | ＞60 | ＞20 | 6 | 30 | 0-25 | 80 |
| Guillén-Solà2017[63] | ＞60 | ＜20 | 3 | 40 | NA | 80 |
| Zhang2016^[64]^ | ＞60 | ＜20 | 4 | 20 | 0-15 | 25 |

Abbreviation:y:Year;d:Day;min:Minute;W:Week
