# Supplementary material for: Effects of transcutaneous neuromuscular electrical stimulation on post-stroke dysphagia: a systematic review and meta-analysis
Source: Front Neurol. 2023 May 9;14:1163045. doi: 10.3389/fneur.2023.1163045 (PMC10203701; doi:10.3389/fneur.2023.1163045)
Supplement: Supplementary file 2 [file Data_Sheet_2.docx]

Supplementary Material

**Effects of transcutaneous neuromuscular electrical stimulation on post-stroke dysphagia： a systematic review and meta-analysis**

# Supplementary Figures

#
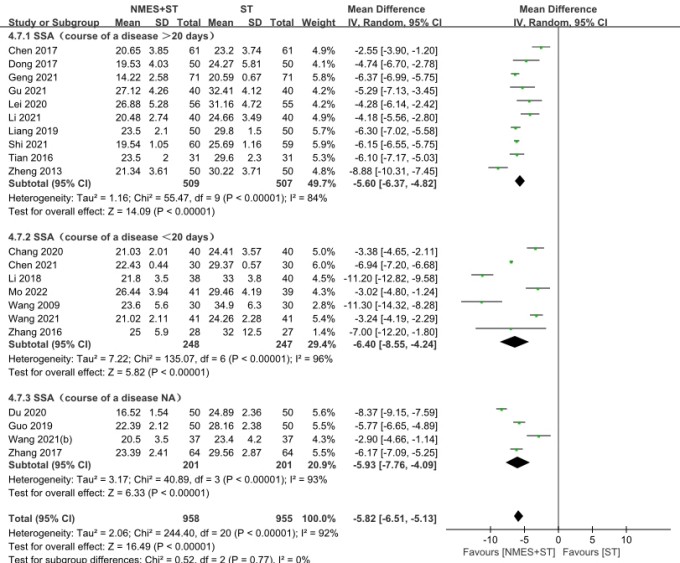


# Supplementary File 1 Subgroup analysis of SSA: Course of a disease


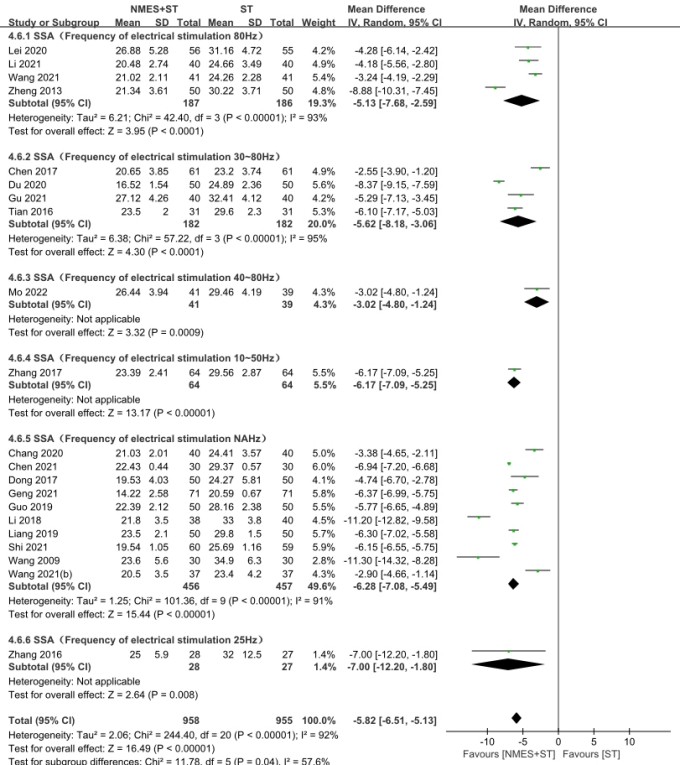


Supplementary File 2 Subgroup analysis of SSA: Frequency of electrical stimulation.


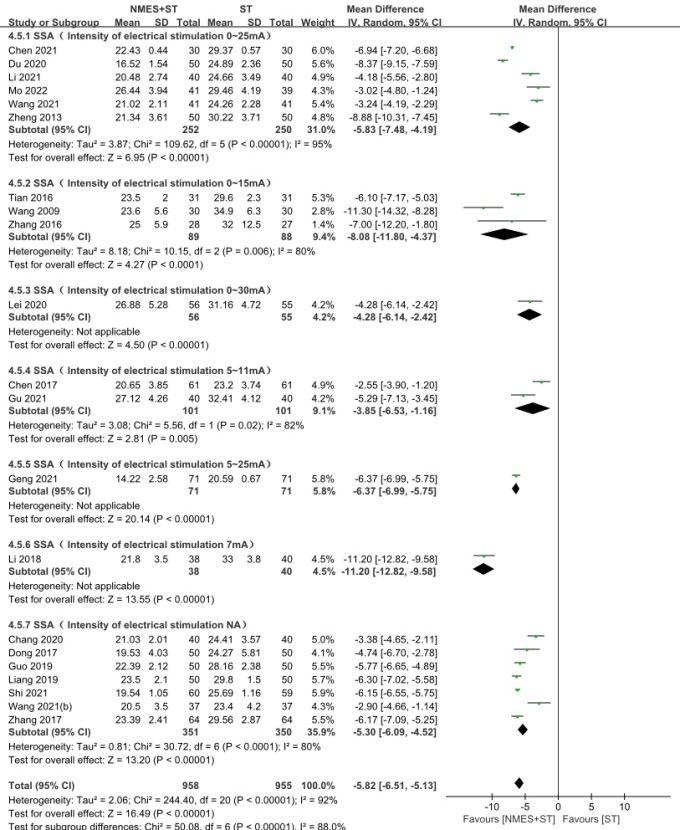


Supplementary File 3 Subgroup analysis of SSA: Intensity of electrical stimulation.


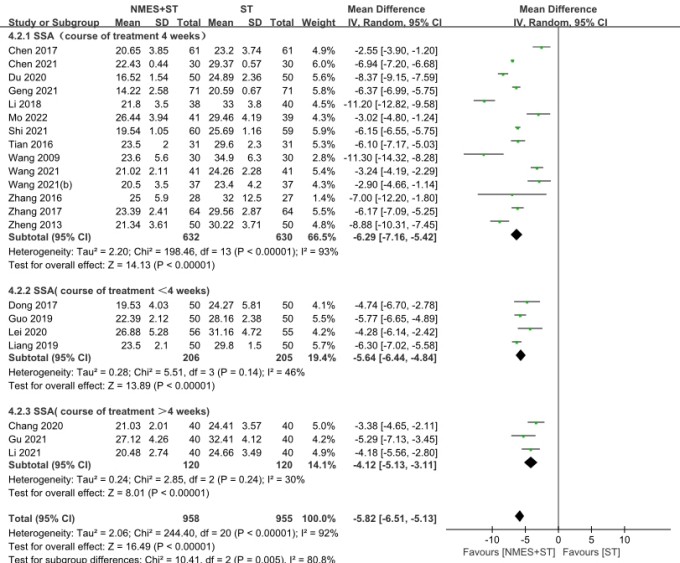


Supplementary File 4 Subgroup analysis of SSA: Course of treatment.


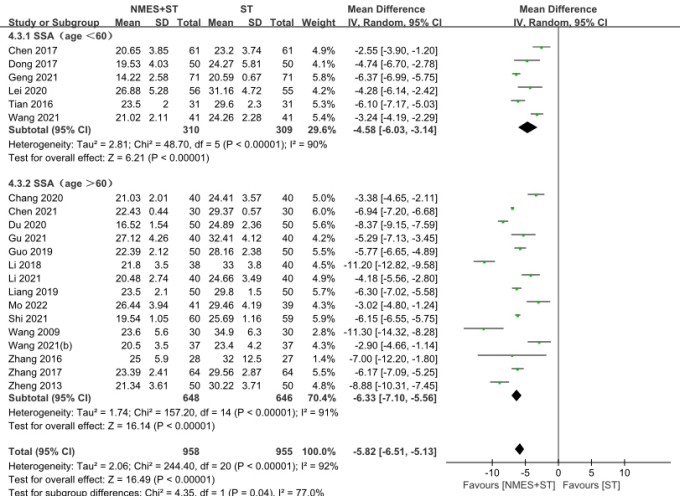


Supplementary File 5 Subgroup analysis of SSA: Age.


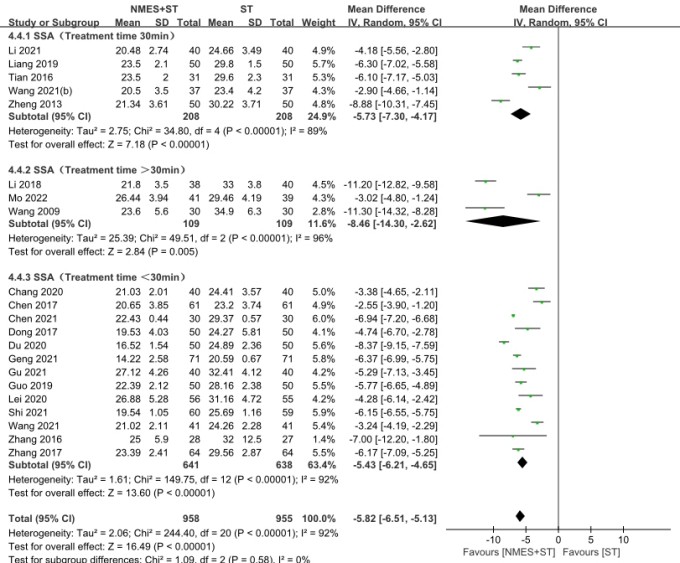


Supplementary File 6 Subgroup analysis of SSA: Treatment time


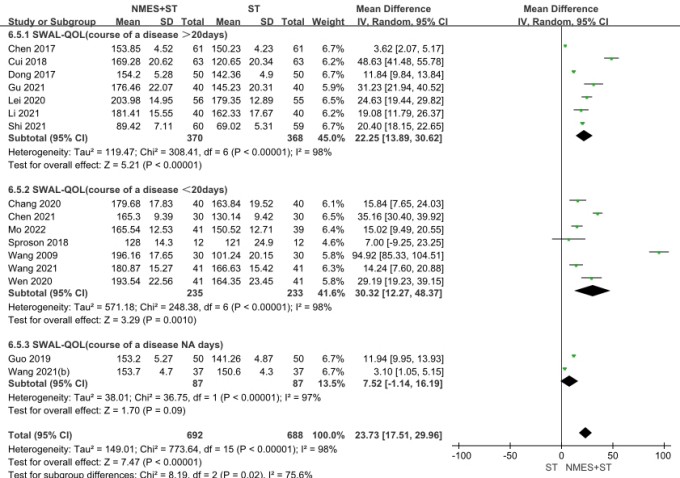


Supplementary File 7 Subgroup analysis of SWAL-QOL: Course of disease.


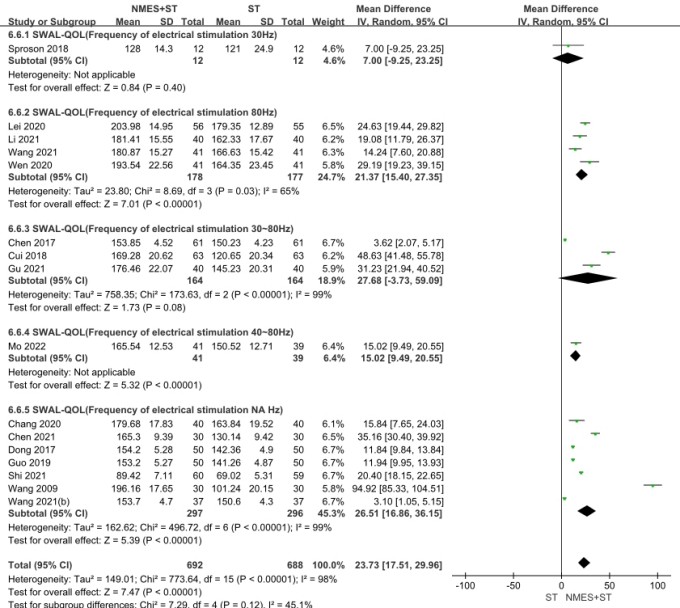


Supplementary File 8 Subgroup analysis of SWAL-QOL:Frequency of electrical stimulation


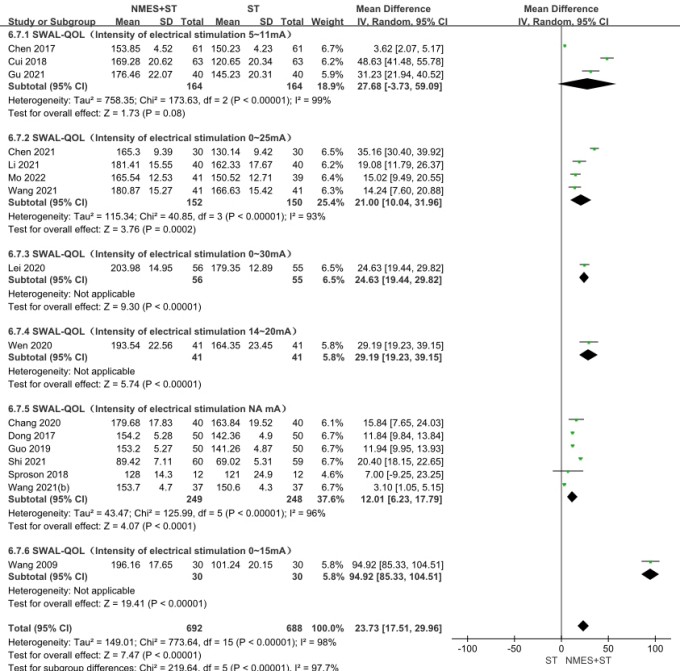


Supplementary File 9 Subgroup analysis of SWAL-QOL:Intensity of electrical stimulation


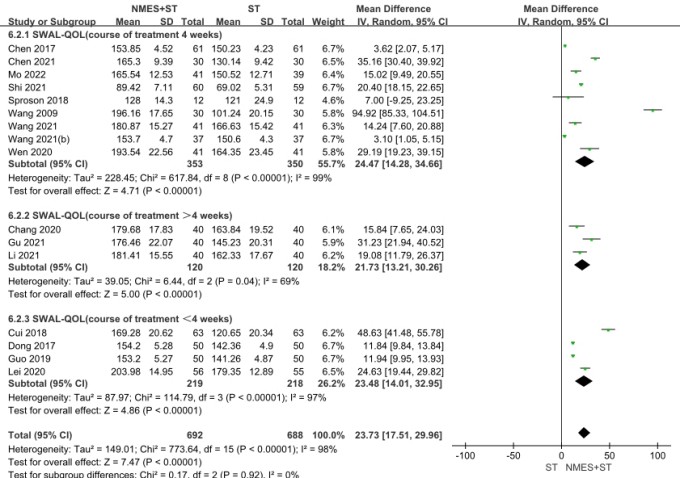


Supplementary File 10 Subgroup analysis of SWAL-QOL:Course of treatment


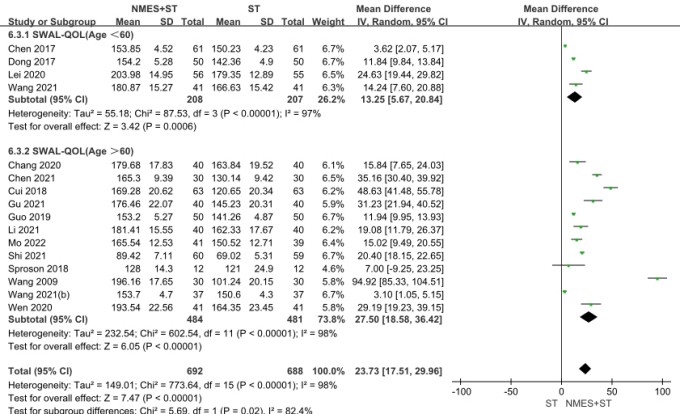


Supplementary File 11 Subgroup analysis of SWAL-QOL:Age


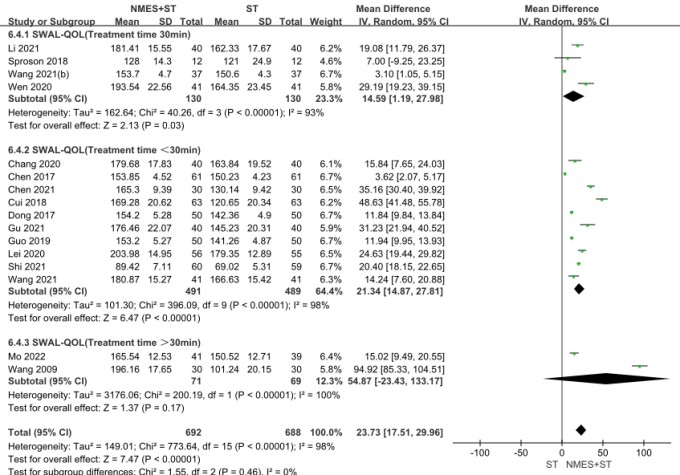


Supplementary File 12 Subgroup analysis of SWAL-QOL:Treatment time


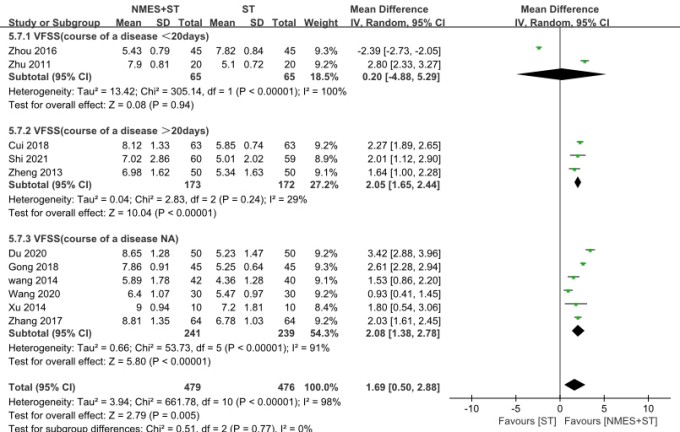


Supplementary File 13 Subgroup analysis of VFSS:Course of a disease


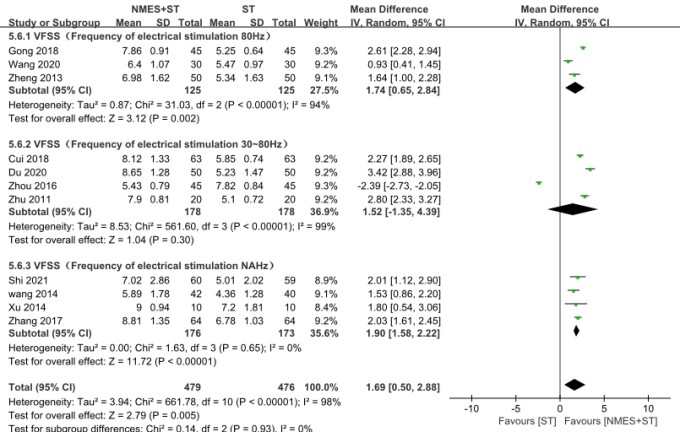


Supplementary File 14 Subgroup analysis of VFSS:Frequency of electrical stimulation


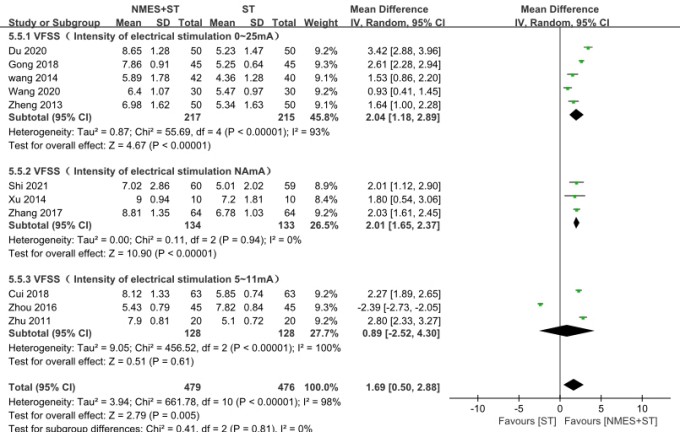


Supplementary File 15 Subgroup analysis of VFSS:Intensity of electrical stimulation


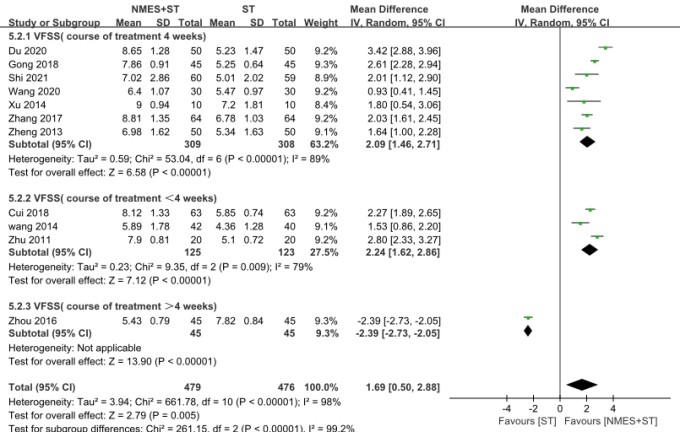


Supplementary File 16 Subgroup analysis of VFSS:Course of treatment


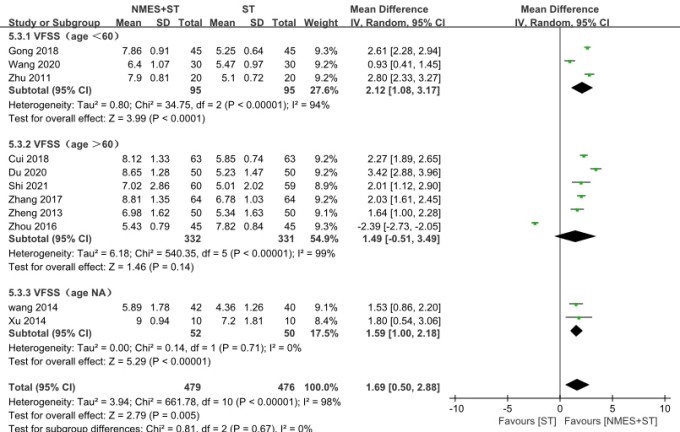


Supplementary File 17 Subgroup analysis of VFSS:Age


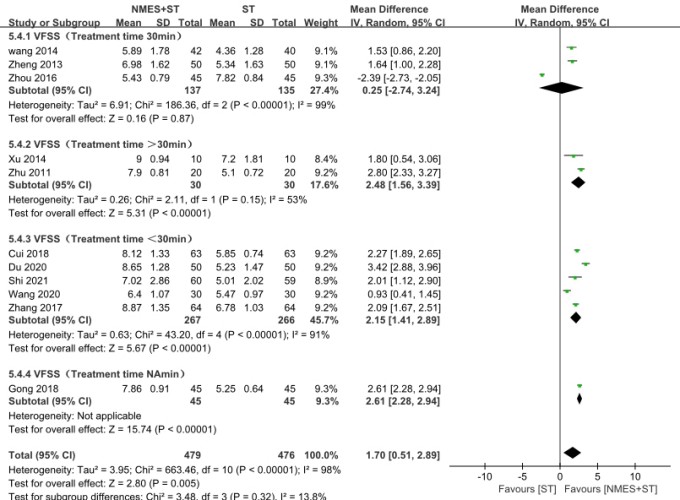


Supplementary File 18 Subgroup analysis of VFSS:Treatment time


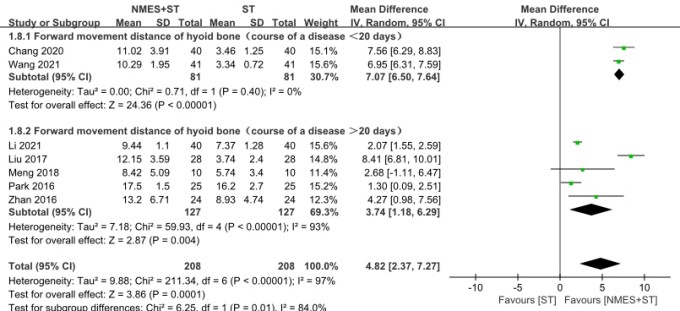


Supplementary File 19 Subgroup analysis of Forward movement distance of hyoid bone:Course of a disease


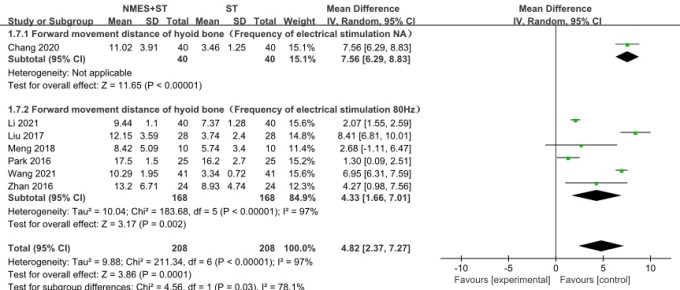


Supplementary File 20 Subgroup analysis of Forward movement distance of hyoid bone:Frequency of electrical stimulation


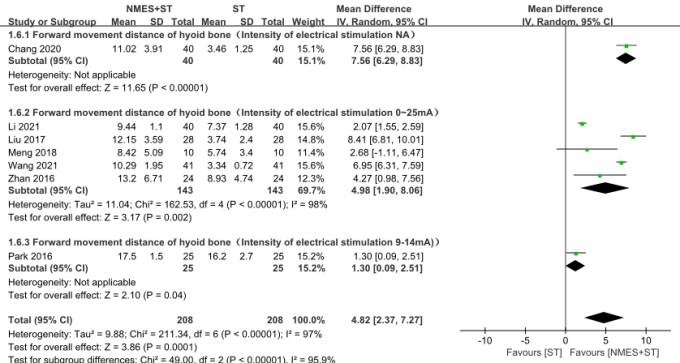


Supplementary File 21 Subgroup analysis of Forward movement distance of hyoid bone:Intensity of electrical stimulation


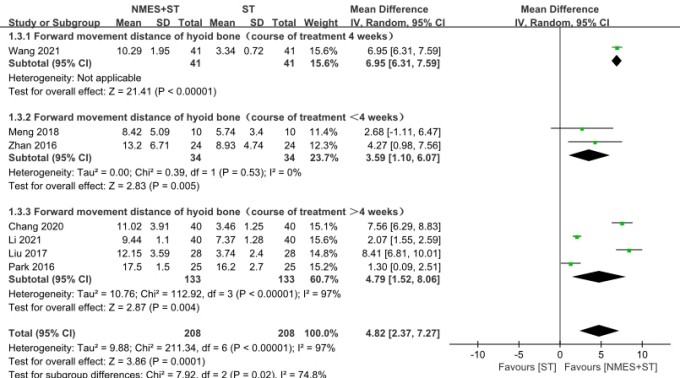


Supplementary File 22 Subgroup analysis of Forward movement distance of hyoid bone:Course of treatment


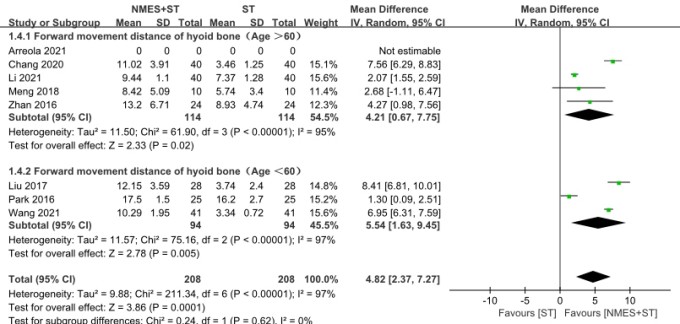


Supplementary File 23 Subgroup analysis of Forward movement distance of hyoid bone:Age


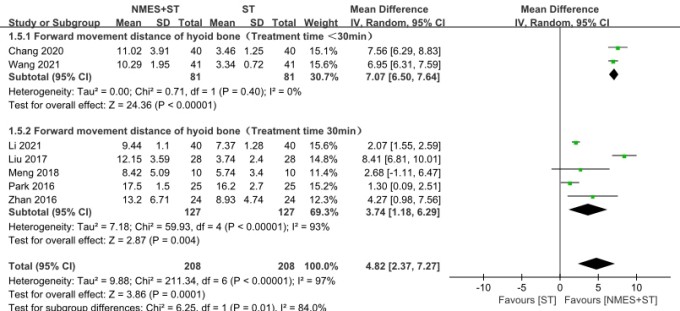


Supplementary File 24 Subgroup analysis of Forward movement distance of hyoid bone:Treatment time


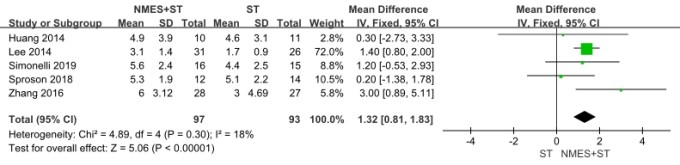


Supplementary File 25 A Forest plot for Functional Oral Intake Scale


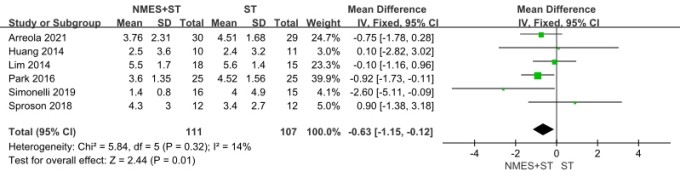


Supplementary File 26 A Forest plot for Penetration-Aspiration Scale


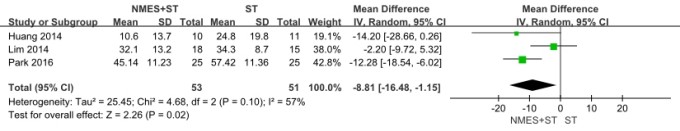


Supplementary File 27 A Forest plot for Functional Dysphagia Scale


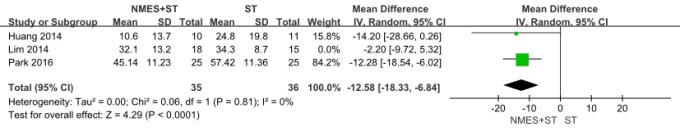


Supplementary File 28 A Forest plot for Functional Dysphagia Scale


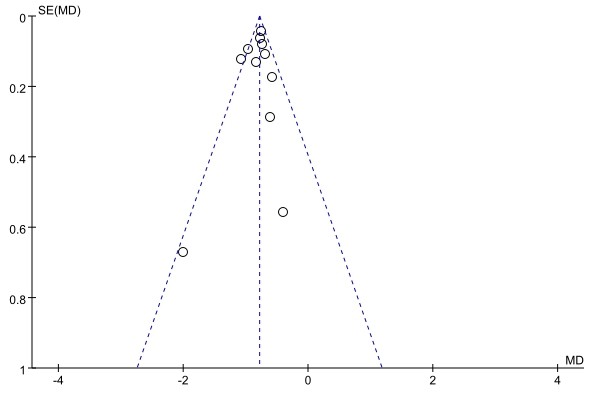
Supplementary Figure 29 Funnel plot for Water Swallow Test
